# The effect of disinfectants and antiseptics on co- and cross-selection of resistance to antibiotics in aquatic environments and wastewater treatment plants

**DOI:** 10.3389/fmicb.2022.1050558

**Published:** 2022-12-13

**Authors:** Daniel Basiry, Nooshin Entezari Heravi, Cansu Uluseker, Krista Michelle Kaster, Roald Kommedal, Ilke Pala-Ozkok

**Affiliations:** ^1^Department of Chemistry, Bioscience and Environmental Engineering, Faculty of Science and Technology, University of Stavanger, Stavanger, Norway; ^2^School of Biosciences, University of Birmingham, Birmingham, United Kingdom

**Keywords:** disinfectants, antiseptics, wastewater treatment plants (WWTP), cross-resistance, antibiotic resistance

## Abstract

The outbreak of the SARS-CoV-2 pandemic led to increased use of disinfectants and antiseptics (DAs), resulting in higher concentrations of these compounds in wastewaters, wastewater treatment plant (WWTP) effluents and receiving water bodies. Their constant presence in water bodies may lead to development and acquisition of resistance against the DAs. In addition, they may also promote antibiotic resistance (AR) due to cross- and co-selection of AR among bacteria that are exposed to the DAs, which is a highly important issue with regards to human and environmental health. This review addresses this issue and provides an overview of DAs structure together with their modes of action against microorganisms. Relevant examples of the most effective treatment techniques to increase the DAs removal efficiency from wastewater are discussed. Moreover, insight on the resistance mechanisms to DAs and the mechanism of DAs enhancement of cross- and co-selection of ARs are presented. Furthermore, this review discusses the impact of DAs on resistance against antibiotics, the occurrence of DAs in aquatic systems, and DA removal mechanisms in WWTPs, which in principle serve as the final barrier before releasing these compounds into the receiving environment. By recognition of important research gaps, research needs to determine the impact of the majority of DAs in WWTPs and the consequences of their presence and spread of antibiotic resistance were identified.

## Introduction

Multi drug resistant (MDR) microorganisms have become a major threat to both global health and economy. The World Health Organization (WHO) has declared that the microbial infections caused by antibiotic resistance bacteria will lead to massive preventable deaths (Collignon, 2017; Morrison and Zembower, 2020). They also estimated that the global economy will suffer over 100 trillion dollars in low- and middle-income countries over the course of the next 30 years due to MDR microorganisms (Collignon, 2017; Morrison and Zembower, 2020).

As MDR microorganisms become more prevalent it is crucial to consider different environments, co-factors involved and sources of emergence, to evaluate the potential threat originating from MDR microorganisms. In this context, disinfectants and antiseptics (DAs) are two significant factors as they are not only widely used in medical and manufacturing sectors but also in private households (Russell, 2013). The recent global pandemic of SARS-CoV-2 has extensively increased the use of DAs as their use was one of the main counter measures against the virus (Usman et al., 2020). This is a potential human health risk as a correlation between DA exposure and resistance to relevant antibiotics has been shown (Kampf, 2018a). DAs are classified as biocides that are widely used and important in industrial and health related applications (Orth, 1998; Dettenkofer and Spencer, 2007; Møretr et al., 2017). In comparison to antibiotics, they normally do not target specific enzymes (Mcdonnell and Russell, 1999; Lachapelle et al., 2013). This makes them good for general purpose application, but bad for treatment of health conditions. DAs however, can serve as a selector for cross-resistance to antibiotics (Varela and Manaia, 2013).

With only a few exceptions, the literature does not consider the correlation between the concentrations of DAs in water bodies, their treatment methods in wastewater treatment plants (WWTP), which receive industrial, hospital and municipal sewage (Karkman et al., 2018), and the potential impact of DAs on the development of cross-resistance to antibiotics.

This review examines the current knowledge on disinfectants and antiseptics and their relationship with the antibiotics. Moreover, it highlights their potential impact on the development of cross- and co-resistance in the aquatic environments with emphasis on wastewater treatment plants. Finally, it provides conclusive evidence on the impact of prioritized DAs on resistance development and gives suggestions for decreasing their discharge loads into receiving waters while making the future of public health the priority.

## Review methodology

This paper provides a systematic literature review and puts the currently available research on DAs in aquatic environments into context. This study discusses and compares the current literature on DA concentration, their fate, removal, and their influence on existing antibiotic resistance from main scientific databases, which included PubMed, Web of Science (ISI), Scopus, and Google Scholar. The search terms used to obtain the literature for this review included groups of DAs, specific compounds, aquatic environments, and cross/co-resistance for antibiotic resistance genes (“antimicrobial resistance” or “antibiotic resistance” or “antimicrobial susceptibility”). Titles and abstracts were screened, followed by the full texts of the papers deemed relevant. Unpublished data was excluded from this review and only publications in English were included. The papers similarities and differences were compared. The classes of disinfectants were chosen based on their relevance for aquatic environments, hence aerial disinfectants were excluded from this review.

## Disinfectants and antiseptics and their mechanism of action

Biocide is a collective term covering all chemical substances used with the intent to inactivate harmful or undesired variety of species which include microorganisms and rodents. Inactivation can be further distinguished into “-static” substances which only inhibit further growth or “-cidal” substances which kill the organism (Mcdonnell and Russell, 1999).

**Figure 1:**
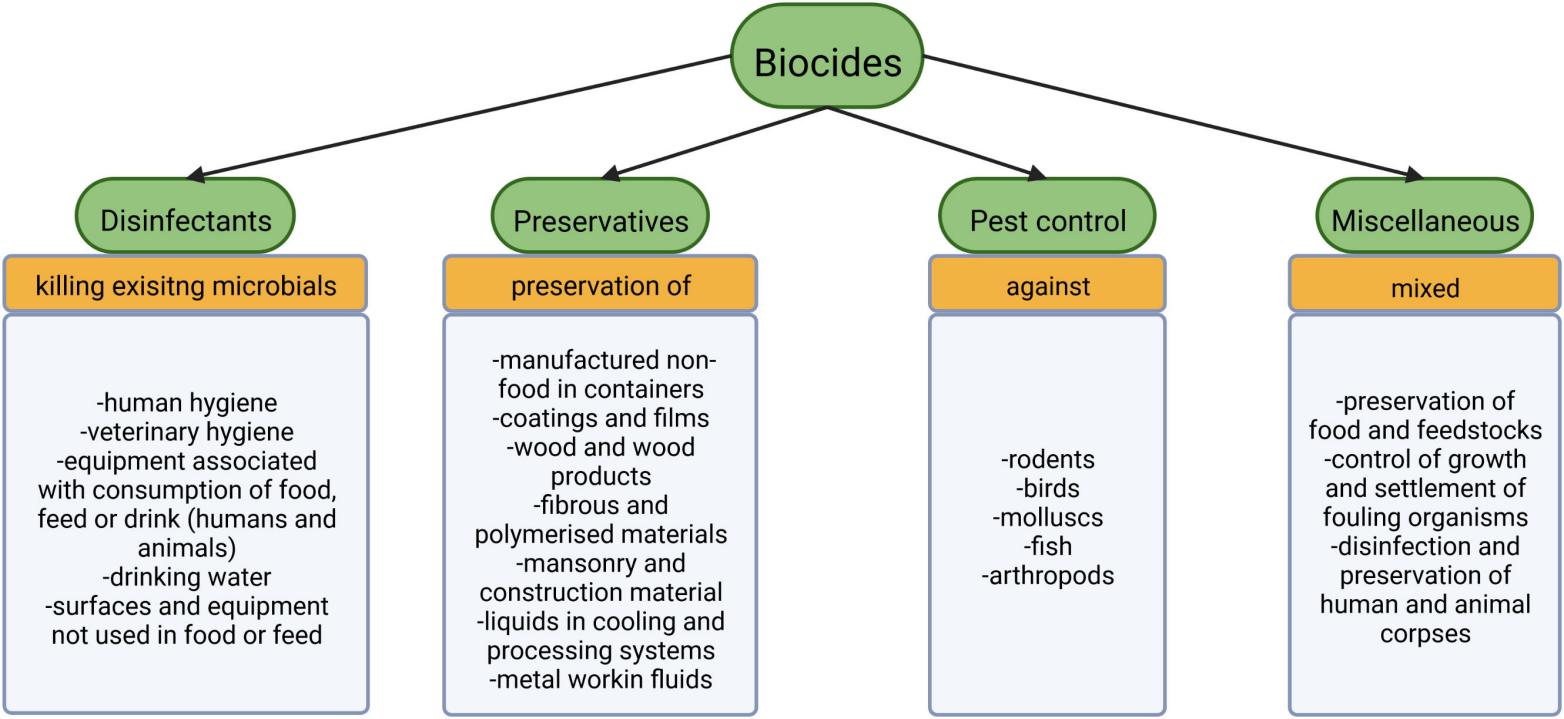
The four big subgroups of biocides according to EU legislation with the associate products (Smith, 2015).

According to EU legislation biocides include four major groups of substances: disinfectants, preservatives, pest control and miscellaneous (Smith, 2015). Disinfectants aim at killing already existing organisms in various fields employed further specified in Figure 1 (Smith, 2015). The preservatives group tries to prolong the time until decay takes place (Smith, 2015). Compounds for pest control serve to poison larger organisms (Smith, 2015), while substances that cannot be categorized as any of the above fall into the miscellaneous group (Figure 1). Antiseptics are non-antibiotic compounds used to prevent or limit infection on living tissues (European Committee for Standardization, 2020). For further details on antibiotics and their spread in WWTPs see for example Barancheshme and Munir (2018) and Uluseker et al. (2021).

Common DAs groups and their targets as well as their mode of action are listed in Table 1 and a table with common representatives and structures (Supplementary Table 1).

**Table 1:** Displayed are DA groups with common representatives and their mode of action.

**Group**	**Function**	**Target**	**Mode of action**	**References**
Alcohols	Antisepsis, disinfection	Cytoplasmic and membrane proteins	Membrane damage, enzyme inhibition, coagulation of cell components	Boyce, 2018
Aldehydes	Disinfection, sterilization	Outer membrane, cell wall	Cross linking of proteins and nucleic acids	Bowes and Cater, 1968; Gorman et al., 1980; Maillard, 2002; Migneault, 2004
Anilides	Antisepsis	Cell membrane, lipid biosynthesis	Inhibition of enoyl-acyl carrier protein reductase	Carey and McNamara, 2014
Biguanides	Antisepsis, disinfection	Cell wall, membrane	Disrupting membrane integrity	Chawner and Gilbert, 1989a,b
Bisphenols	Antisepsis, disinfection	Cell membrane, proteins	Loss of membrane integrity, protein coagulation	Walia et al., 2017
Chelating agents	Antisepsis, disinfection	Cell membrane	Chelating metal ions, loss of membrane integrity	Finnegan and Percival, 2015
Halophenols	Antisepsis	Cell membrane, Lipid biosynthesis	Inhibition of enoyl-acyl carrier protein reductase (FabI)	Jang et al., 2008; Larras et al., 2020
Heavy metal derivatives	Antisepsis, disinfection	Thiol groups in proteins, PMF*, DNA bases	Cytological changes, K+ leakage, intercalation	Chappell and Greville, 1954; Rosenkranz and Rosenkranz, 1972; Modak and Fox, 1973; Schreurs and Rosenberg, 1982; Feng et al., 2000; Dibrov et al., 2002
Halogen-releasing agents	Antisepsis, disinfection	Thiol groups in proteins	Oxidation to disulphides causing inhibition and modification	Dyer et al., 2019
Phenols and cresols	Disinfection	Cell membrane, PMF, proteins	Loss of membrane integrity, protein coagulation, inhibition electron transport chain	Judis, 1965, 1966; Denyer et al., 2004
Peroxygens	Disinfection, sterilization	Thiol groups in proteins	Oxidation to disulphides causing inhibition and modification	Small et al., 2007
Quaternary ammonium compounds	Antisepsis, disinfection	Cell membrane lipids; cell wall	Leakage of cell components through membrane damage	Hugo and Longworth, 1965; Gilbert and Moore, 2005

PMF*, proton motive force.

While broad-spectrum antibiotics exist, antibiotics tend to have specific intracellular targets when compared with DAs. DAs, unlike antibiotics, have a broader and unspecific toxicity and target membrane proteins, cell wall, nucleic acids and thiol groups in proteins (Denyer and Stewart, 1998).

The outer layer of protection in most bacteria is the cell wall, which encircles the plasma membrane (Maillard, 2002). The cell wall consists of peptidoglycan chains crosslinked by short peptides. Gram-negative bacteria have an outer membrane in addition to the cell wall (Maillard, 2002). Certain DAs bind lipid components of the cell wall or replace cationic components which can cause deformation of the cell wall and the underlying plasma membrane, weakening the cell wall integrity and causing leakage of components.

The cell or plasma membrane exists in most cells in the form of a lipid double layer and separates the external environment from the cell interior. It consists mainly of phospholipids and contains proteins of varying functionality e.g., transport proteins redox enzymes (Maillard, 2002). Across the cell membrane the proton-motive force (PMF) enables active transport of specific molecules and sustain essential cellular processes (Le et al., 2021). Through disruption of the cell wall and membrane various DAs cause loss of structural integrity and leakage of cell components such as K^+^, nucleotides and amino acids (Hugo and Longworth, 1965; Jensen, 1975; Chawner and Gilbert, 1989b,a).

**Figure 2:**
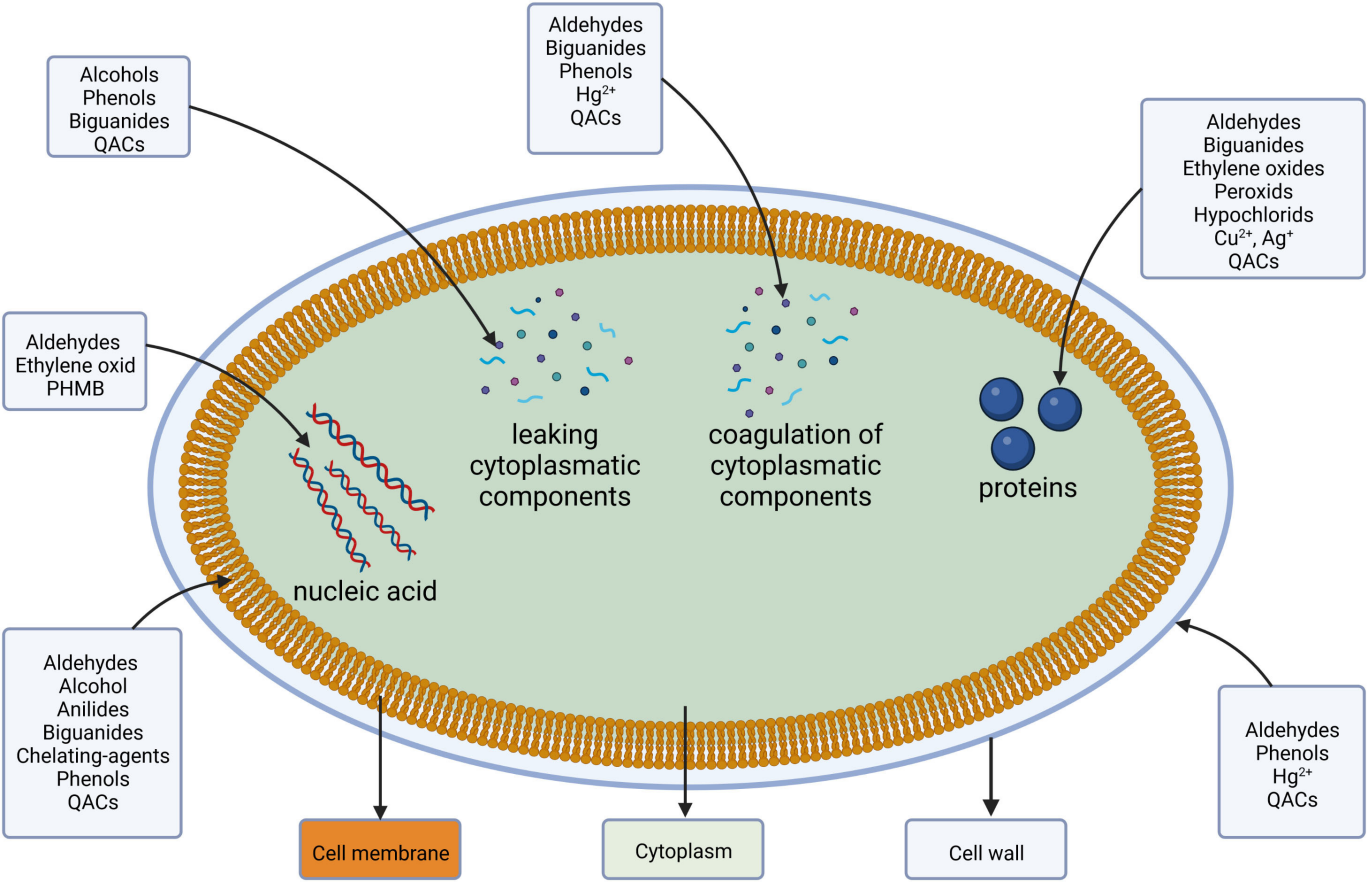
Disinfectants and antiseptics and their target location and components.

In the cytoplasm, a direct interaction between the cellular machinery and DAs occurs. Binding of or reacting with DNA causes inhibition of protein syntheses, reactive components interact specifically with a variety of proteins and cause coagulation (Maillard, 2002). A visualization of the different target sides is shown in Figure 2 followed by a more detailed look into their respective groups' resistance mechanism.

### Alcohols

As broad-spectrum antimicrobial agents, alcohols are very effective against vegetative bacteria (including mycobacteria), viruses, and fungi (Stawarz-Janeczek et al., 2021). While they are not considered to be sporicidal, they can inhibit sporulation and spore germination. Their main application is hard-surface disinfection and skin antisepsis. They are not used for sterilization (Elekhnawy et al., 2020). Ethyl alcohol (ethanol) and isopropyl alcohol (isopropanol) are the two most widely used alcohols (Morton, 1950) applied at low levels in clinical settings for many years (Boyce, 2018). Another often used alcohol is bronopol which is used in pharmaceutical and cosmetic products (Shepherd et al., 1988). Depending on the active agent and target microorganisms, isopropyl alcohol is more potent against bacteria (Coulthard and Sykes, 1936; Mcdonnell and Russell, 1999), and ethyl alcohol is more effective against viruses (Klein and Deforest, 1983; Mcdonnell and Russell, 1999). To increase the efficiency of alcohol's antimicrobial activities, they are produced in combination with low levels of other biocides or excipients. For instance, the presence of excipients like emollients reduces the alcohol evaporation time. As another example, chlorhexidine improves the alcohol products' effectiveness significantly by remaining on the target surface after alcohol evaporation (Bush et al., 1986). The optimal concentration for alcohol antimicrobial activity is between 60 and 90% (Boyce, 2018).

Alcohols cause several different reactions in microbial cells. Short chained alcohols for example can cause reduced cross-linking of peptide glycan, precipitation of nucleic acids and leakage of the latter and small molecules (Ingram and Buttke, 1985). Bronopols mechanism of action on the other hand is attributed to its ability to cross-link sulfohydrid-groups of dehydrogenase enzymes on the cell surface (Legin, 1996).

### Aldehydes

Aldehydes are a significant broad-spectrum disinfectant group from which the most critical agents are glutaraldehyde, formaldehyde and o-phthalaldehyde (Fraud et al., 2001). Glutaraldehyde (GA) serves as an important DA in different areas such as disinfection and sterilization of hospital equipment and environments, animal husbandry and as a general disinfectant of surface areas (Juncker, 2015). It has broad spectrum activity against bacteria, spores, fungi and viruses (Migneault, 2004). Formaldehyde is effective against bacteria, spores, fungi and viruses (Graham and Barger, 1936; Tilley, 1945; Korukluoglu et al., 2006) and it is used either in liquid or vapor form. In 2011, the U.S. Department of Health and Human Services stated that formaldehyde is a known human carcinogen (U.S. Department of Health and Human Services PHS, 2021). O**-**phthalaldehyde is a broad-spectrum disinfectant with activity against bacteria, viruses and mycobacteria as well as spores but less efficient compared to other aldehydes (Alfa and Sitter, 1994; Gregory et al., 1999; Walsh et al., 1999, 2001).

The efficiency of aldehydes is attributed to their ability to effectively cross-link outer wall components, enzymes and other proteins, disabling their function (Bowes and Cater, 1968; Gorman et al., 1980; Maillard, 2002; Migneault, 2004). As aldehydes exist in a polymeric, aqueous solution the alkylation responsible for the cross-linking happens through chemical reactions depending on, for example, pH-value of the disinfectant solution (Migneault, 2004). Aldehydes react with nucleophilic functional groups such as amine, thiol, phenol and imidazole, for example in the amino acids lysine (Bowes and Cater, 1968), tyrosine, tryptophan, phenylalanine (Hopwood et al., 1970), histidine and cysteine (Lopachin and Gavin, 2014). In addition to cross-linking aldehydes also cause irreversible changes to protein structures through alkylation (Bowes and Cater, 1968; Migneault, 2004). This happens mainly through reactions with sulfhydryl, hydroxyl, amine and carboxyl groups (Bowes and Cater, 1968; Hopwood et al., 1970). Aldehydes can also alkylate the amino groups of purines and pyrimidine bases which can result in mutations (Loshon et al., 1999).

### Anilides

Anilides are based on the two structures, salicylanilide and diphenylurea. Triclocarban (TCC), based on diphenylurea, is the most extensively used anilide, widely applied in various personal care products and household consumables including, soaps, shampoos, and toothpaste, since 1957 (Musee, 2018). This broad-spectrum antimicrobial agent is particularly active against gram-positive bacteria.

A growing body of literature describes the adverse consequences of persistent TCC residues in the environment and their potential impact on plants, animals, humans, and microorganisms (Yun et al., 2020). Endocrine disruption, bioaccumulation, acute/chronic toxicity, and possible antibiotic resistance are the main consequences (Halden, 2014). It has been reported that exposure to 200 μg/L of TCC causes growth retardation and reduced fecundity in some marine species (Han et al., 2016).

Anilide's mechanism of action is due to its protonophoric activity and ability to discharge parts of the proton-motive force (Kratky and Vinsova, 2011). This causes a lower extracellular protein production and may cause autolysis (Kratky and Vinsova, 2011).

### Biguanides

Biguanidines used as antiseptics and disinfectants contain at least two biguanidine elements in their structure (Kathuria et al., 2018). Popular biguanides like chlorhexidine have been in use and under research since the mid-20th century (Davies et al., 1954).

Chlorhexidine is a broad-spectrum antiseptic and disinfectant. It is used as an antiseptic for medical and veterinarian antisepsis, oral disinfection and hand scrubs (Lowbury and Lilly, 1960; Holloway et al., 1986; Kuruvilla and Kamath, 1998; Traor et al., 2000; Gomes et al., 2001). Chlorhexidine works against gram positive and negative bacteria, fungi and is spore- and mycobacteriostatic (Davies et al., 1954; Beeuwkes, 1958; Ortegón et al., 2017).

Alexidine is an antimicrobial of the biguanide class, and is used in antiseptics, antiplaque solutions (Gjemro et al., 1973) and in contact lens solutions (Rosenthal et al., 2006). Alexidines works by altering membrane permeability.

Another effective biguanide is the mixture called polyhexamethylene biguanides (PHMB) consisting of polymeric biguanides with varying end groups such as amine, cyanoguanide and guanidine (Allen M. J. et al., 2006). The European chemicals agency considers it to be safe to use for swimming pool disinfections and surface cleaning (ECHA, 2017).

Biguanides linking mechanism of action is the alteration of membrane permeability and the leakage of intracellular cell components (Hugo and Longworth, 1965; Jensen, 1975; Chawner and Gilbert, 1989b,a). The hexamethylene chain in chlorhexidine is essential for its effectiveness. Varying the length of the chain can lead to reduced antimicrobial efficiency with hexamethylene being the optimal length (Davies et al., 1954). Biguanides cause coagulation at concentrations higher than the minimum inhibitory concentration (MIC). Coagulation is not considered as the reason for the cell death (Hugo and Longworth, 1965) but rather for a reduction in leakage which is attributed to the coagulated proteins plugging the porose membrane (Hugo and Longworth, 1965).

PHMB, in addition to the general biguanide mechanism, strongly binds nucleic acids and consequently interferes with the expression of proteins and causes changes in the transcriptional profile (Allen et al., 2004; Allen M. J. et al., 2006). Recent research showed that PHMB selectively condensed bacterial chromosomes (Chindera et al., 2016).

### Bisphenols

Bisphenols are sporostatic, hydroxy-halogenated compounds derived from two phenolic groups conjunct by numerous bridges (Sasatsu et al., 1995). Despite their broad-spectrum activity, they are not efficient against gram-negative bacteria, like *E. coli* and *P. aeruginosa*, and molds (Lloyd et al., 1988). Hexachlorophene was an extensively used bisphenol that was mainly applied as an antiseptic agent in personal care products (Marzulli and Bruch, 1981) until concerns over its neurotoxicity led to it being banned in the US in 1970s (Heidler and Halden, 2009). Experiments on *Bacillus megaterium* showed that hexachlorophene adsorbs to the membrane, causing leakage, protoplast lysis and respiration blockage by disruption of the PMF (Silvernale et al., 1971). While the leakage occurs mostly at higher concentrations (Joswick et al., 1971), at MIC for hexachlorophene it binds tightly to the membrane and inhibits uptake of amino acids and respiration. Like other lipophilic acids, its main mechanism is the inhibition of the PMF (Levin and Freese, 1977).

### Chelating agents

Chelating agents, for example ethylenediaminetetraacetic acid (EDTA), work against Gram- positive and -negative bacteria, yeast, amoeba and fungi (Finnegan and Percival, 2015). ETDA is used as a contact lens disinfectant, on medical equipment, for wound care (Finnegan and Percival, 2015), and has excellent properties for biofilm removal (Percival et al., 2005; De Almeida et al., 2016).

Metal chelating agents like EDTA cause destabilization of the membrane through removal of metal ions like Mg^2+^ and Ca^2+^-ions (Vaara, 1992). These metal ions stabilize the negatively charged phospholipids in the outer layer. Through the removal of these ions, lipopolysaccharides (LPS) from the cell wall are rapidly released and lost (Leive, 1965). It was proposed that chelating agents like EDTA, *N*-hydroxyethylethylenediamine-*NN'N'*-triacetic acid (Walsh et al., 2003) and to a lesser extent nitrilotriacetic acid (Haque and Russell, 1974) serve as a potentiating agent, as they destabilize the outer membrane and create easier access for other compounds.

### Halogen-releasing agents

Halogen-releasing agents are extensively used as antiseptics, disinfectants, and preservatives. The most used halogens are chlorine and iodine-based compounds applied in clinical settings (McDonnell, 2009).

Iodine is a broad-spectrum antimicrobial effective against bacteria, mycobacteria, fungi, protozoa, and viruses (Maksym and Gmur, 2020). This agent causes cell death through passive diffusion through the cell membrane and intracellular oxidation of proteins, nucleotides, and fatty acids (Lepelletier et al., 2020).

Chlorine-releasing agents (CRAs) are widely used for hard-surface disinfection. Hypochlorites including sodium hypochlorite (liquid form) and calcium hypochlorite (solid form) are the most used disinfectants in this group (Bennett et al., 2015). In water, sodium hypochlorite is ionized to the hypochlorite ion (${\text{OCl}}_{2}^{-}$) at equilibrium with the hypochlorous acid (HOCl). Depending on the pH, chlorine can predominate as HOCl or ${\text{OCl}}_{2}^{-}$. The former predominates in pH between 4 and 7, and the latter in pH above 9 (Bloomfield, 1996). A hybrid system of sodium chlorite and mandelic acid has been found to be effective as an antiseptic (Mcdonnell and Russell, 1999).

CRAs biocidal activity is attributed to their function as strong oxidizing agents. Targets are various chemical groups in proteins, especially sulfhydryl groups but they are also known to cause cellular lesions (Bloomfield, 1996).

### Halophenols

Halophenols have a similar base mechanism to that of the non-halogenated phenols. Through the halogenation the phenol reaches higher activity than phenols, but the water solubility is reduced (Zhao and Chen, 2016).

Triclosan (TCS) is a synthetic broad-spectrum antimicrobial that is efficient against both gram-positive and gram-negative bacteria. It is also effective against some fungal species, parasites, and protozoa species (Bhargava and Leonard, 1996; Fang et al., 2010). Since 1962, it has been used in a wide range of products and applications in many countries (Allmyr et al., 2008; Iyer et al., 2017). It is bacteriostatic at concentrations lower than 0.1 mg/L, and exhibits bactericidal activity against numerous species, including *E. coli* and *Streptococci*, at concentrations above 2.0 mg/L. Besides the antibacterial properties, triclosan may have anti-inflammatory effects against inflammatory skin conditions (Alfhili and Lee, 2019). However, possible antibiotic resistance has been reported because of TCS exposure (Halden, 2014).

The mode of action of triclosan in bacterial cytoplasm includes the disruption of lipid biosynthesis through inhibition of the enzyme enoyl-acyl carrier protein reductase (FabI) (Larras et al., 2020). However, the exact mechanism that leads to the switch from bacteriostatic to bactericidal action is still unknown (Yasir et al., 2020). Triclosan exposure resulted in upregulation of multidrug resistance genes in *S. aureus*. These genes are involved in coenzyme transport and the downregulation of genes for virulence factors, energy metabolism and of several enzymes involved in lipid metabolism (Jang et al., 2008).

Chloroxylenol (PCMX) is a halophenol widely used as antiseptic for skin (e.g., hand soap) and as disinfectant for abiotic surfaces. It has been commercially available in Dettol handwashing products since 1920 and is now frequently used in over-the-counter products used in domestic and hospital environments (Mcdonnell and Russell, 1999; Yasir et al., 2020). After the ban of triclosan and triclocarban in disinfectant formulations in 2016 (Sreevidya et al., 2018) there was a sudden increase in consumption of PCMX as an alternative. Although PCMX is a broad-spectrum antimicrobial which is very effective against bacteria, fungi, algae and viruses, there are still some molds, and the gram-negative bacteria for example *P. aeruginosa*, that are very resistant to it (Bruch, 1996; Sreevidya et al., 2018). Due to phenolic nature of PCMX's, interactions between the hydroxyl groups of this agent and cytoplasmic membrane proteins can destroy the microbial membranes leading to cell death (Hamilton, 1970; Mcdonnell and Russell, 1999).

The phenolic compound PCMX works by causing loss of cell membrane integrity and leakage of cell components (Hamilton, 1970; Mcdonnell and Russell, 1999; Walia et al., 2017). Denyer and Stewart (1998) proposed the idea that this causes an autocidal chain reaction inside the cell. The loss of cytoplasmatic components leads to the initiation of degradative enzymes and the creation of free radicals. In addition to the loss of cell membrane integrity, PCMX causes coagulation of cytoplasmic components through interactions between the hydroxyl–OH groups of this agent and proteins (Hamilton, 1970; Mcdonnell and Russell, 1999; Walia et al., 2017).

In terms of ecotoxicity, PCMX has chronic effects on the red blood cells of aquatic organisms, even at levels as low as 4 μg/L (Capkin et al., 2017). This compound can also disrupt gene expression and cell tissue structures (Kasprzyk-Hordern et al., 2009).

### Heavy metal derivates

Heavy metals appear in different oxidative states and their antimicrobial activity varies depending on the oxidative state of the metal. Copper, mercury, and silver are the most used metals as DA.

Copper (Cu) compounds are mainly sulfate, citrate or nitrate salts releasing the active Cu^2+^-ion. Even though it has a wide range of bactericidal and viricidal activity (Borkow and Gabbay, 2005; Noyce et al., 2007), it is mainly used as an algaecide or fungicide (Borkow and Gabbay, 2005). Copper compounds are also utilized as preservative in the wood industries (Brient et al., 2020).

Because of coppers redox chemistry it takes part in the formation reactive oxygen species (Peña et al., 1999). These radicals can cause major harm to the cell through oxidation of membrane lipids, proteins (Peña et al., 1999). The assumption that Cu ions cause DNA damage through Fenton-like oxidation process is increasingly doubted as molecules like glutathione chelate copper (Macomber et al., 2007).

Mercury (Hg) compounds are today mainly used in the form of organo derivates like nitromersol, thimerosal, merbromin and phenylmercuric nitrate. Organomercury are used less often in the west as disinfectants since they are considered toxic (Gilpin et al., 2021). Thimerosal is still used as an antiseptic in multi vaccine doses, especially in the third world countries (Geier et al., 2015).

Silver (Ag) compounds and silver nanoparticles (AgNPs) have a wide variety of usage. They are used on medical devices to prevent the growth of biofilm, in treatment of burn wounds and as an additional disinfectant in water systems of pools and hospitals (Silver, 2003; Mijnendonckx et al., 2013; Stout and Yu, 2014; Norman et al., 2017).

Silver ions interfere with the PMF and respiratory chain enzymes and cause the leakage of K^+^ ions (Chappell and Greville, 1954; Schreurs and Rosenberg, 1982; Dibrov et al., 2002) and act as a DNA intercalating agent based around the Ag^+^-ion and its high reactivity (Rosenkranz and Rosenkranz, 1972; Modak and Fox, 1973; Feng et al., 2000). The main targets are thiol groups causing cytological changes, intercalating in nucleotide chains, inhibiting cell division and inactivating proteins (Rosenkranz and Rosenkranz, 1972; Modak and Fox, 1973; Feng et al., 2000).

### Phenols and cresols

Phenolic-type antimicrobial agents are used as disinfectants to control the growth of microorganisms. These compounds have different inhibitory effects against diverse bacteria, fungi, and viruses (Yarley et al., 2021). Due to their fungicidal and antiseptic properties, they are used in many different industrial products and processes including; pharmaceuticals, textiles, plasticizers, pulp and paper, pesticide manufacturing, the wood industry, detergent application, and metallurgic industries (Santana et al., 2009). It has been suggested that their membrane-active properties contribute to their overall activity (Davidson and Branden, 1981; Mcdonnell and Russell, 1999).

de León et al. (2010) indicated that cell membranes of both gram-positive and gram-negative bacteria are vulnerable to phenolic antimicrobial agents cause leakage of cell components such as K^+^, nucleotides and amino acids. Higher phenol concentrations result in coagulation of cytoplasmic constituents leading to irreversible cellular damage (Hugo, 1992). Phenolic compounds because of their lipid-soluble properties able to dissipate the PMF (McLaughlin and Dilger, 1980; Kasianowicz et al., 1984; Mitchell, 2011).

The potential toxicity and negative impacts of phenolic compounds on the environment have been investigated in a large body of literature, suggesting that some of these compounds (particularly chlorophenols) are highly toxic, estrogenic and carcinogenic for some aquatic organisms (Ferreira Guedes and Leitão, 2012; Catherine et al., 2016). Additionally, phenolic compounds can inhibit microorganisms present in activated sludge, and therefore disrupt treatment performance in wastewater treatment facilities (Salaudeen et al., 2019).

### Peroxygens

This class of disinfectants has been in use since the 19th century and represents environmentally friendly disinfectants as water is their only by-product.

Hydrogen peroxide is used in the food industry for processing, handling and production, hard-surface disinfection in medical institutions for critical equipment (Agency USEP, 1993), in distribution of drinking water, skin disinfection (1–6%) and in the preservation of paper additives (EU, 2015; Murphy and Friedman, 2019). It is also used in the disinfection of wastewater (Gulyas et al., 1995; Lin et al., 1999). It is a broad-spectrum disinfectant against bacteria, viruses, and fungi (Baldry, 1983).

Peracetic acid is a more potent disinfectant than hydrogen peroxide and works against mycobacteria and spores (Baldry, 1983). It is used in the food industry, human hygiene, and disinfectant for surfaces, the medical sector and drinking water (Agency USEP, 1993; European Commission, 2016). Peracetic acid is also used for the disinfection of wastewater (Liberti and Notarnicola, 1999).

Performic acid's activity spectrum includes bacteria, spores, viruses fungi and mycobacteria (Rutala and Weber, 2001). The food and medical sector use performic acid as a disinfectant (Gehr et al., 2009) and it is also used in wastewater disinfection (Gehr et al., 2009; Chhetri et al., 2019). Performic acid is unstable and is normally produced on site in a solution containing performic acid, formic acid, hydrogen peroxide and water (Gehr et al., 2009).

Peroxygens mode of action is based on free radical oxidation of enzymes and protein thiol groups (Denyer and Stewart, 1998) breaking down proteins, nucleic acids and membranes leaving, innocuous and non-toxic end products (Liberti and Notarnicola, 1999; Gehr et al., 2009; Chhetri et al., 2019). In the case of nucleic acids this can induce single and double strand breaks independent of cell type (Dizdaroglu and Jaruga, 2012). To better identify the effects of peroxygen exposure, Small et al. (2007) looked at the transcriptome during peroxygen exposure. *P. aeruginosa* was exposed to hypochlorite, hydrogen peroxide and peracetic acid. In addition to the upregulation of general stress genes as protection against peroxygen disinfectants, hydrogen peroxide and hypochlorite each caused a more specific response (Small et al., 2007). When exposed to hydrogen peroxide, DNA repair genes were upregulated (Small et al., 2007). Hypochlorite exposure caused oxidative phosphorylation, electron transport and proteins actively transporting hexose molecules to be downregulated (Small et al., 2007). Organic sulfur transporter genes and membrane proteins in general were upregulated (Small et al., 2007).

### Quaternary ammonium compounds

The structure of Quaternary ammonium compounds (QAC) constitutes of an ammonium with four substitutes and a small anion with further distinction into mono-, di-, tri- and polymeric structures. The biocidal activity mostly originates from the length of the alkyl chains and can be adapted to target different microorganisms (McBain et al., 2004).

Benzalkonium chloride (BAC) is a broad-spectrum disinfectant lethal to gram-positive and negative bacteria, lipophilic viruses, fungi- and algae static (Beveridge et al., 1998; Obłak et al., 2021). BAC is a mixture of varying alkyl group chain length. It is used in hand scrubs, surface disinfectant, wound and skin antiseptic and as a wood preservative (Report, 2012).

Cetrimide is a composition from tetradonium bromide, cetrimonium bromide and laurtrimonium bromide. It is used as a wound and skin antiseptic and in combination with chlorhexidine is used to clean medical instruments (Tripathi, 2019).

The biostatic and cidal activity of QACs is mainly attributed to displacement of outer membrane associated cations such as Ca^2+^ and subsequent intercalation into the membrane (Gilbert and Moore, 2005). As a consequence, a leakage of low molecular cellular components takes place causing autolysis (Davies et al., 1968; Bonesvoll and Gjermo, 1978; Tischer et al., 2012). Cetyltrimethylammonium bromide, part of the QAC mixture cetrimide, intercalates into DNA and causes precipitation (Allen G. C. et al., 2006), as well as other QACs (Zinchenko et al., 2004). The chain length of the side group polymer influences the antimicrobial effectiveness of QACs (Dizman et al., 2004; Lu et al., 2007).

## Occurrence of disinfectants and antiseptics in aquatic environments, and their fate in wastewater treatment plants

Disinfectants and antiseptics ultimately reach WWTPs. As these WWTPs have not been designed to remove these components, and due to the current inability of wastewater treatment setups a significant fraction of these compounds reaches the receiving water bodies due to the discharge of WWTP effluent. Their presence in natural water cycles represent a risk to human health and the ecosystem. The current section covers the occurrence of these compounds in water bodies including surface waters, WWTP influent and effluent, together with the observed removal potential, the existing situation in typical WWTPs. The occurrence data have been categorized according to the source and summarized in Tables 2–4. Additionally, due to the concern of impact of co-occurrence of DA and antibiotics on antibiotic resistance, this section provides data on the co-occurrence of disinfectants, antiseptics, and antibiotics in WWTPs. The concentrations of DA and antibiotics were listed in this section, when detected in co-occurrence, however it is important to note that their co-occurrence is variable depending on different conditions including season, sampling location, type of WWTP and types of DA and antibiotics that occur together.

**Table 2:** Occurrence of disinfectants in wastewater treatment plants (WWTPs) and wastewater (WWs).

**Compound**			**Concentration (μg/L)**	**Source**	**Country**	**References**
Alcohols	Ethyl sulfate		1.4–74	Raw WW	Spain	López-García et al., 2020
Aldehydes	Glutaraldehyde		Up to 0.0131	Raw WW	South Korea	Lim et al., 2013
			0.0167	WWTP Effluent		
	Formaldehyde		Up to 0.3462	Raw WW		
			Up to 0.3211	WWTP Effluent		
Anilides	Triclocarban		6.7	Raw WW	U.S.	Halden and Paull, 2005
			0.0047–0.0762	Raw WW	China	Sun et al., 2016
			0.0276–0.109	WWTP Effluent		
Biguanides	Chlorhexidine		1.305	Raw WW	Sweden	Östman et al., 2017
Chelating agents	Ethylenediaminetetraacetic acid (EDTA)		5.7–330	Raw WW	Austria	Clara et al., 2012
			6.5–310	WWTP Effluent		
			585	Raw WW	UK	Gardner et al., 2013
	Nitrilotriacetic acid (NTA)		71–830	Raw WW	Austria	Clara et al., 2012
			Up to 410	WWTP Effluent		
Halogen-releasing agents	AOX		1300–302,500	Pharma. WWTP Effluent	China	Xie et al., 2017
	Iodine		10,000–30,000	Raw LCD WW	South Korea	Lee et al., 2009
			Up to 100	Raw WW	France	Wiest et al., 2018
			5-40	WWTP Effluent	US	Drewes et al., 2001
Halophenoles	Chloroxylenol		0.228–27.832	Raw WW	UK	Kasprzyk-Hordern et al., 2009
	Triclosan		0.087–15.729			
Heavy metals derivatives	Mercury		0.066	Raw WW	UK	Gardner et al., 2013
			0.7 (±8%)− 3.8 (±49%)		Italy	Carletti et al., 2008
			1.26		Sweden	Östman et al., 2017
	Copper		79	Raw WW	Greece	Hargreaves et al., 2018
			17		Brazil	
			59		France	
			73		US	
			76		UK	
			62		Australia	
			32		Italy	
			10			
			10			
			38			
			61			
			71		Wisconsin, U.S.	
			70			
			107			
			53		Sweden	Östman et al., 2017
	Silver		< 0.15–2.1	Raw WW	Norway	Polesel et al., 2018
			0.06–2.6	WWTP Effluent	U.S.	Shafer et al., 1998
			0.49	Raw WW	Sweden	Östman et al., 2017
Phenols and cresols	Phenol		0.74 ± 0.08	Raw WW	Spain	Llompart et al., 2002
			121 ± 50	Coking WWTP Effluent	China	Zhou et al., 2005
	Cresols	p-Cresol	1.1 ± 0.1	Raw WW	Spain	Llompart et al., 2002
			32 ± 110	Coking WWTP Effluent	China	Zhou et al., 2005
		o-Cresol	81 ± 60			
	Phenolic compounds		0.000643–0.30583	Raw WW	Italy	Spataro et al., 2019
Quaternary ammonium compounds (QACs)	QACs		0.002–0.072	WWTP Effluent	Sweden	Östman et al., 2017
	Benzalkonium chloride (BACs)		Up to 170	Raw WW	Austria	Kreuzinger et al., 2007
			Up to 40.1	WWTP Effluent		

**Table 3:** Occurrence of disinfectants in surface waters, drinking/tap waters and drinking water treatment plants (DWTPs).

**Compound**		**Concentration (μg/L)**	**Source**	**Country**	**References**
Aldehydes	Glutaraldehyde	0.3–1.4	DWTP Effluent	South Korea	Kang and Shin, 2016
Anilides	Triclocarban	0.008– 1.119	River	India	Vimalkumar et al., 2018
		0.360		South Africa	Lehutso et al., 2017
Chelating agents	Ethylenediaminetetraacetic acid	07–4.0	River	Germany	Giger et al., 2006
	Nitrilotriacetic acid				
Halogen-releasing agents	Iodine	0.4–212	River	Europe and US	Moran et al., 2002
		0.1–0.4	Tap water	China	Gong and Zhang, 2013
Halophenols	Chloroxylenol	< 0.03–0.358	River	UK	Kasprzyk-Hordern et al., 2008
		0.06–1.2		Indonesia	Dsikowitzky et al., 2016
		>500	Industrially impacted estuarial areas	Global	Thomas et al., 1999
	Triclosan	Up to 0.001	River	Germany	Bester, 2005
		Up to 0.347		China	Zhao et al., 2009
		0.88–8.72		South Africa	Lehutso et al., 2017
		14.7	River	Taiwan	Shen et al., 2012b
		Up to 0.2	Tap water		
Heavy metals derivatives	Mercury	0.0001–0.02	River	Global	Watras et al., 1995
	Silver	0.00247–0.0698	River	Germany	Wimmer et al., 2019
Phenols and cresols	Phenolic compounds	Up to 0.458	River	Germany	Bolz et al., 2001
		0.652–3.3	Lake	China	Zhong et al., 2010

**Table 4:** Occurrence of disinfectants in hospital wastewater.

**Compound**		**Concentration (μg/L)**	**Country**	**References**
Aldehydes	Glutaraldehyde	500–4,000	France	Jolibois et al., 2002
Biguanides	Chlorhexidine	85–1,940	Japan	Matsushima and Sakurai, 1984
Halogen-releasing agents	AOX	200–1,700	France	Wiest et al., 2018
Heavy metals derivatives	Mercury	21 ± 1	Mexico	Pérez-Alvarez et al., 2018
		0.04–2.6	Europe	Kümmerer, 2001

### Alcohols

Alcohols are biodegradable compounds that are quickly metabolized in wastewater. A study investigating six pharmaceutical WWTPs in China found that alcohols were only detected in the influents, however, alcohol degradation residues are detected in WWTPs (Luo et al., 2019). In a study conducted in Spain, the stable ethanol residue ethyl sulfate was found in wastewater in ranges between 1.4 μg/L and 74 μg/L (López-García et al., 2020). Among the alcohols, isopropanol is extensively used in pharmaceutical fields leading to the production of large amounts of wastewater containing this compound (Cui et al., 2019; Yan et al., 2022). However, isopropanol is a valuable organic solvent that usually is recovered from industrial wastewater through separation processes (Zhou et al., 2019). According to a recent literature review by Verovšek et al. (2022) the concentration of alcohol residues in surface waters and wastewater has not been examined in any major reviews.

### Aldehydes

Glutaraldehyde (GA) has been detected in urban sewage networks, surface waters, and even drinking water (Boillot and Perrodin, 2008; Wang et al., 2011). GA values found in drinking water samples ranged from 0.3 to 1.4 μg/L (Dizman et al., 2004). In hospital wastewater it was measured at a concentration of 0.50 mg/L (Kang and Shin, 2016) with peak concentration up to 4 mg/L can be seen in hospital effluents (Kang and Shin, 2016). Formaldehyde is a common chemical in various applications, and it was the 25th most produced chemical in the USA in 1999 (Edwards et al., 1999). It can be found in very high concentrations (12,900 mg/L) in specific industrial wastewater (Hidalgo et al., 2002). GA and formaldehyde were detected in the effluent and/or influent of 11 different livestock WWTPs in South Korea, along with some antibiotics including chlortetracycline, oxytetracycline, and trimethoprim (Lim et al., 2013). Up to 13.1 and 16.7 ng/L of GA were detected in the influent and effluent WWTP samples, respectively. For formaldehyde the concentrations detected in the influent and effluent were up to 346.2 and 321.1 ng/L, respectively, and chlortetracycline was detected at up to 70,866.5 ng/L and 4,516.5 ng/L in the influent and the effluent, respectively. Oxytetracycline and trimethoprim were only detected in the influent in concentrations up to 12,171.9 ng/L and 0.4 ng/L, respectively (Lim et al., 2013).

Biodegradability studies conducted on glutaraldehyde showed variable results, which ranged from 83% in 5 days to 98% in 20 days for different samples. Moreover, different studies showed that lower concentrations of GA (<2 mg/L) in WWTPs resulted in higher biodegradability of this compound (Leung, 2001). The variability of the test results was attributed to the shortcomings of different test methods. However, the overall analysis of results led to the conclusion that glutaraldehyde was readily biodegradable in the water environment (Leung, 2001). Evaluation of microbial community metabolism revealed that microorganisms quickly mineralize glutaraldehyde first to glutaric acid and then to CO_2_ under aerobic conditions (Leung, 2001; Langenhoff, 2011). Additionally, glutaraldehyde has been shown to prefer to stay in the aquatic phase (Langenhoff, 2011), however, the GA that remains unmetabolized after biological treatment can be removed by sorption on the biomass (Leung, 2001). Dilution to decrease GA concentrations is impractical due to the large volumes of required water, however, sodium bisulfite in a 2 to 3 molar ratio was found to be the most effective chemical deactivation method for an aqueous glutaraldehyde solution before entering the sewage facilities (Jordan et al., 1996).

Formaldehyde in wastewater is removed by several mechanisms which include adsorption, biological and chemical oxidation methods (Eiroa et al., 2005; Jarusutthirak et al., 2012; Bellat et al., 2015; Talaiekhozani et al., 2016; Yuan et al., 2017). Formaldehyde in municipal wastewaters originates from industrial manufacturing of common products including paper, leather, and glass. It is biodegradable under anaerobic and aerobic conditions, however above 250 mg/L it becomes toxic to microorganisms. Formic acid is an intermediate during formaldehyde degradation, which is also known to be easily biodegradable (Jarusutthirak et al., 2012).

### Anilides

Global consumption of products containing triclocarban (TCC) has led to detectable concentrations of this compound in raw and treated wastewaters as well as in the receiving waters (Shen et al., 2012a,b; Taweetanawanit et al., 2018). Data mainly from China and the US showed TCC to be detectable in surface and drinking waters (Yun et al., 2020). The average concentration in US raw wastewater was measured as 6.7 μg/L (Halden and Paull, 2005). These data were significantly higher than those observed in India and South Africa at 1.119 and 0.360 μg/L, respectively (Lehutso et al., 2017; Vimalkumar et al., 2018). Sun et al. (2016) studied the occurrence and fate of various pharmaceuticals and personal care products in three different WWTPs in Xiamen, China, and observed the co-occurrence of TCC (Inf: 4.7–76.2 ng/L, Eff: 27.6–109 ng/L) with three different antibiotics including oxytetracycline (Inf: 8.6–230 ng/L, Eff: below the method detection level (BLD)-51.4 ng/L), sulfamethoxazole (Inf: BLD−95.2 ng/L, Eff: BLD−22.4 ng/L), and tetracycline (Inf: BLD−189 ng/L, Eff: BLD−37.6 ng/L) in the influent and effluent of the plants.

Observed removal efficiency of TCC in WWTPs ranged from 11.4 to 97% mainly due to adsorption onto sludge (Yun et al., 2020). This significant disparity probably derives from differences in overall treatment design, inlet loading and concentrations, process operational conditions, and hydraulic and solids retention time (Ying and Kookana, 2007; Lehutso et al., 2017; Armstrong et al., 2018). A study conducted at a large U.S. activated sludge WWTP found that TCC is not completely removed during wastewater treatment and approximately 3% residual is discharged with the effluents (Heidler et al., 2006).

Lozano et al. (2013) suggested some TCC degradation occurred during nitrification-denitrification in the secondary treatment stage, possibly due to reductive dehalogenation. On the other hand, Wang et al. (2020) reported the biological nutrient removal processes to be bioprocesses relevant for nitrogen and phosphorus removal can significantly be inhibited by long-term exposure to TCC concentrations of 100 μg/L or higher (Wang et al., 2020). Due to its high hydrophobicity [log K_OW_ = 4.3; (Information NC for B, 2004)] TCC will strongly partition to activated sludge and potentially be released upon use as fertilizer on farmlands (Heidler et al., 2006; Wang et al., 2020). Ultraviolet (UV) oxidation is considered as a putative mechanism for degradation of TCC (Ali et al., 2011).

### Biguanides

Chlorhexidine was detected in hospital wastewater in Japan at concentrations ranging from 0.085 to 1.94 mg/L (Matsushima and Sakurai, 1984). In Sweden the average concentration reported of chlorhexidine for influent wastewater was 1,305 ng/L and in treated effluent, concentrations of five antibiotics including ciprofloxacin, clarithromycin, erythromycin, metronidazole and trimethoprim were detected in addition to an average chlorhexidine concentration of 28 ng/L (Östman et al., 2017). From the detected antibiotics, trimethoprim was found in all samples in the range of 10–130 ng/L, and the highest concentration was found for erythromycin, which was 350 ng/L (Östman et al., 2017).

Östman et al. (2018) studied detailed mass flows and removal efficiencies of chlorhexidine in three Swedish WWTP. The results of this study suggested that chlorhexidine removal is associated mainly with adsorption onto sludge particles. No biodegradation was observed for chlorhexidine in the studied WWTPs. The maximum observed removal efficiency from wastewater was 98% *via* sorption onto sludge, while the rest ended up in the digested sludge (Östman et al., 2017). Over 99% removal was obtained in a drinking water treatment plant in Spain using advanced treatment processes, including ultrafiltration, reverse osmosis, and granular activated carbon (Boleda et al., 2011).

### Bisphenols

Hexachlorophene was widely used in personal care products during the 1970s, and its presence was observed in sewage water and sludge (Neal, 1973; Heidler and Halden, 2009). However, the US Food and Drug Administration (USFDA) banned it over concerns neural damage in infants (Kimbrough, 1973). Heidler and Halden (2009) reported the influent and effluent wastewater concentrations of this compound as <0.11 ppb and <0.02 ppb, respectively. Additionally, their results suggested that the compound was removed from wastewater by adsorption to the active sludge (Sasatsu et al., 1995).

### Chelating agents

Ethylenediaminetetraacetic acid (EDTA) and nitrilotriacetic acid (NTA) are chelating agents found at μg/L levels in WWTPs and surface waters. The concentrations of EDTA and NTA measured in the influent and effluent of a WWTP in Austria were between 5.7 and 330 μg/L and 6.5–310 μg/L for EDTA, and 71–830 μg/L and n.d. (not detected)-410 μg/L for NTA, respectively (Clara et al., 2012). The concentrations of EDTA with a mean value of 585 μg/L and three antibiotics namely erythromycin, ofloxacin, and oxytetracycline with mean concentrations of 2 μg/L, 0.18 μg/L and 3.6 μg/L, respectively, were observed in the influent of sixteen WWTPs in the UK (Gardner et al., 2013). EDTA and NTA were among the most abundant organic compounds found in the river Rhine in Germany with concentrations between 0.7 and 4.0 μg/L with an average value of 1.8 μg/L (Giger et al., 2006).

Different studies showed that NTA could be removed up to 90% through biological wastewater treatment, while removal efficiency of EDTA was only between 15 and 23% (Clara et al., 2012). As both compounds are very hydrophilic with log K_OW_ values of −2.6 and −3.8 (National Center for Biotechnology Information, 2004a,b), respectively, they are not expected to accumulate in the sludge. However, high hydrophilicity together with poor biodegradability results in poor EDTA removal during wastewater treatment. Therefore, in order to reduce its discharge into receiving waters, it is crucial to control the sources and to apply advanced treatment processes like ozonation and adsorption onto activated carbon (Margot et al., 2015).

### Halogen-releasing agents

When chlorines are added to wastewater they react quickly with biological materials and produce various organo-chlorinated compounds known as adsorbable organic halogens (AOX). AOX are known to be lipophilic, persistent, and toxic in aquatic environments (Kümmerer, 2001; Emmanuel et al., 2004). Detectable levels of AOX compounds in treated effluents from different WWTPs in China was reported to be in the range of 1.3–302.5 mg/L (Xie et al., 2017). AOX are not readily biodegradable but typically, can be removed to between 34 and 89% in biological treatment processes, mainly by adsorption to the activated sludge (Bryant et al., 1992; Xie et al., 2017).

The raw wastewater collected from treatment plant at an LCD film polarize manufacture in South Korea contained iodine concentrations of 10 to 30 mg/L (Lee et al., 2009), which was also detected in surface waters from 129 North American and European rivers and tap water from four Chinese cities in concentrations of 0.4–212 μg/L (Moran et al., 2002) and 0.1–0.4 μg/L (Gong and Zhang, 2013), respectively. The organic iodine concentration of 5–40 μg/L in treated effluents from different WWTPs in the U.S. shows that this compound is not completely removed during treatment (Drewes et al., 2001). Laboratory experiments by Drewes et al. (2001) showed removal rates of organic Iodine under aerobic conditions were negligible. While anoxic conditions led to partial removal of organic iodine (around 20%), the highest biodegradation of 57.3 % was observed under anaerobic conditions (Drewes et al., 2001). These results are not surprising as organic halogen compounds are well known not to be transformed aerobically but remediated anaerobically *via* reductive dehalogenation under anaerobic conditions.

A study in France reported the co-concentrations of AOX and three antibiotics involving ciprofloxacin, sulfamethoxazole, and vancomycin, in hospital and urban raw wastewaters. In this study, AOX, ciprofloxacin, sulfamethoxazole, and vancomycin were detected in range concentrations (μg/L) of 200–1,700, 4.6–179, 0.9–26, and 0.06–7.4, in hospital raw wastewater and up to 100, up to 0.15, 0.04–1.5, and < Limit of quantification, in urban raw wastewater, respectively (Wiest et al., 2018).

### Halophenoles

The extensive use of Chloroxylenol (PCMX) in domestic and hospital environments has contributed to levels of up to 65 μg/L in urban wastewater treatment plants (Choi and Oh, 2019). A comprehensive monitoring study of pharmaceuticals and personal care products in two rivers in the UK revealed that all samples contained PCMX at a concentration ranging from <0.03 to 0.358 μg/L (Kasprzyk-Hordern et al., 2008) Rivers in greater Jakarta city, Indonesia, contained PCMX of 0.06 and 1.2 μg/L (Dsikowitzky et al., 2016). The concentrations found in this study could be related to nearby sources which include potential leaching form solid waste, WWTPs treating municipal and industrial waste originating from manufactures of plasticizers, paper and flame retardants (Dsikowitzky et al., 2016). Extreme levels above 500 μg/L were reported in one industrially impacted estuarine area, these levels exceed the acute and chronic toxicity thresholds for some aquatic species (Thomas et al., 1999).

Conventional WWTPs can remove PCMX from wastewater through the activated sludge process with an efficiency of up to 99% (Kasprzyk-Hordern et al., 2009). An examination of the PCMX's occurrence and removal in a WWTP in Baltimore US influent and effluent concentrations of 0.4 and 0.08 μg/L, respectively. Moreover, biodegradation test results presented in the same work showed 60% biotransformation of PCMX after 21 days of incubation, which was increased to 80% after additional 50 days of incubation (Yu et al., 2006).

Triclosan (TCS) concentrations in domestic sewage ranged from 0.3 to 12.5 μg/L (Abbott et al., 2020). This antimicrobial and its known transformation product, methyl triclosan (MeTCS), were detected in the range of <3–10 ng/L and 0.3–10 ng/L, respectively, in surface water samples from the river Ruhr in Germany (Bester, 2005). Data from China showed that TCS was present at concentrations between 0.6 and 347+-12 ng/L in the Liuxi, Zhujiang, and Shijing rivers (Zhao et al., 2009). Another study from Taiwan investigated the presence of this compound in various samples, including tap water, treated household drinking water, bottled water, and river water finding TCS levels of up to 0.2, 0.13, 0.1, and 14.7 μg/L, respectively (Shen et al., 2012b). The reported TCS concentration of river water samples in South Africa were generally lower (0.88 and 8.72 μg/L) (Lehutso et al., 2017).

TCS, has been detected in Swiss WWTP effluents at concentrations ranging from 42 and 213 ng/L, which results in TCS in the receiving waters between 11 and 98 ng/L (Singer et al., 2002). The fate of TCS during wastewater treatment has been studied in four different WWTPs in China (Zheng et al., 2020b) were the average influent and effluent TCS concentrations were found to be 397.1 and 8.0 ng/L, respectively. This study found that more than 97% of the TCS was removed in different treatment processes including modified and carousel oxidation ditch and modified A2/O. Their results indicated that while TCS was removed from the wastewater 36.4–49% of the compound was transferred onto the sludge, therefore posing a possible ecological risk and thus, treatment is needed before application onto land used for agriculture. A study by Lozano et al. (2013) indicated that TCS concentrations decreased in the secondary and in the nitrification/denitrification processes with removal efficiencies of 10.4 and 22.6%, respectively, while presence of methyltriclosan (MeTCS), a transformation product of TCS, indicated biotransformation in the nitrification/denitrification process (Lozano et al., 2013). Guerra et al. (2019) found that biological treatment, including facultative and aerated lagoons, were efficient methods for the removal TCS from wastewater, and that there is a strong correlation between TCS removal and organic nitrogen removal.

The cooccurrence of TCS and PCMX with five antibiotics including trimethoprim, sulfamethoxazole, chloramphenicol, erythromycin-H_2_O, and metronidazole were reported in influent and effluent of two WWTPs in UK. Among these antibiotics, trimethoprim had the highest mean concentration of 2,192 and 2,925 ng/L in the influents of each WWTPs, and the mean levels of TCS and PCMX in the same influents were 87 and 228 ng/L in one WWTP, and 15,792 and 27,832 ng/L in the other WWTP, respectively (Kasprzyk-Hordern et al., 2009).

### Heavy metals derivatives

The concentrations of heavy metal derivatives used as DAs that are found in municipal wastewater vary over three orders of magnitudes between ng/L and μg/L levels (Margot et al., 2015; Cervantes-Avilés et al., 2019). Discharge of heavy metals such as mercury (Hg), copper (Cu) and silver nanoparticles (AgNPs) from sources including dental practices, hospitals, agricultural sites, and landfill leachate is high (Wang et al., 2004; Li et al., 2013).

Mercury (Hg) and Copper (Cu) are commonly detected in municipal wastewater and WWTPs (Hargreaves et al., 2018). The average co-concentrations of Hg and Cu and three antibiotics (erythromycin, ofloxacin, oxytetracycline) detected in the influents of several WWTPs in UK were reported as 0.066, 76, 2.0, 0.18, 3.6 μg /L, respectively (Gardner et al., 2013). The detected concentration of Hg in the wastewater from a hospital in Toluca, Mexico, was 21 ± 1 μg/L (Pérez-Alvarez et al., 2018). The average concentration of Hg in European hospital wastewaters varied between 0.04 and 2.6 μg/L (Kümmerer, 2001). In Italy, the average influent Hg concentration in five WWTPs ranged between 0.7 and 3.8 μg/L (Carletti et al., 2008). The globally detected Hg concentrations in surface waters ranged from 0.1 to 20 ng/L, with most values under 5 ng/L (Cossa and Fileman, 1991; Watras et al., 1995; Mastrine et al., 1999). Carletti et al. (2008) reported the typical Hg removal efficiencies of five WWTPs to be between 57 and 92% by sorption onto the sludge.

Domestic inputs were found to be the main sources of Cu entering urban WWTPs, mainly due to corrosion in domestic plumbing systems (Merkel et al., 2002). Hargreaves et al. (2018) reported Cu concentrations in the WWTP influents from different countries including Greece, Italy, Brazil, France, US and the UK. The measured concentrations were seen to vary between 10 and 107 μg/L, details of which are given in Table 2. Moreover, the same study showed that maximum removal efficiency of Cu in studied WWTPs (mainly activated sludge process) was 94% (Hargreaves et al., 2018).

Silver is released into the environment in the form of dissolved ionic Ag^+^ and AgNP (Kaegi et al., 2013). Ag^+^ concentration in the influent wastewater of two Norwegian WWTPs were measured as <0.15–2.1 μg/ L by Polesel et al. (2018). The influent and effluent Ag^+^ concentration was measured in five WWTPs in the Wisconsin (US) area by Shafer et al. (1998). The results suggested that the Ag^+^ removal efficiency was above 94%, mainly *via* adsorption with effluent Ag^+^ concentration of between 0.06–2.6 μg/L. AgNPs were detected in the effluent of seven WWTPs in Germany. In this study AgNP concentrations were measured at the discharge points of these WWTP in the River Isar (Germany), and these ranged from 2.47 to 69.08 ng/L (Wimmer et al., 2019). However, due to rapid dilution and fast adsorption into the river's suspended sediments, the concentration was stable at around 1–2 ng/L until the next discharge point (Wimmer et al., 2019).

Average concentrations of Ag (0.49 μg/L), Hg (1.26 μg/L), and Cu (53 μg/L) together with antibiotics (ciprofloxacin, erythromycin, clarithromycin, trimethoprim, metronidazole) in an average concentration ranging 0–100 ng/L were detected in the influent of several WWTPs in Sweden (Östman et al., 2017).

### Phenols and cresols

Phenolic compounds are common in ecological water samples. Data from the influent of an urban WWTP in Spain showed levels of 0.74 ± 0.08 and 1.1 ± 0.1 μg/L for phenol and p-Cresol, respectively (Llompart et al., 2002). Whereas water samples from a coking WWTP contained phenol, p-Cresol, and o-Cresol in high concentrations of 131.8 ± 0.11, 51.2 ± 0.09, and 17.2 ± 0.06 mg/L for untreated samples, effluents contained only 0.121 ± 0.05, 0.032 ± 0.11, and 0.081 ± 0.06 mg/L, respectively (Zhou et al., 2005). Phenolic compounds are found in surface waters. The highest observed concentration in five rivers and streams in south-west Germany was 0.458 μg/L (Bolz et al., 2001). In the third biggest lake in China, Taihu, concentrations ranged 0.652 – 3.3 μg/L (Zhong et al., 2010). The occurrence of six antibiotics (amoxicillin, ciprofloxacin, tylosin, erythromycin, sulfamethoxazole and chlortetracycline) and four phenolic compounds (bisphenol A, 4-nonylphenol, nonylphenol mono- and di-ethoxylate) were investigated in the inlets and outlets in four WWTPs in Rome, Italy. The maximum average antibiotic concentration was found for chlortetracycline as 2,976.19 ng/L in the influent, in which the average concentrations of phenolic compounds were 305.83, 6.43, 27.55, and 229.09 ng/L for bisphenol A, 4-nonylphenol, nonylphenol mono- and di-ethoxylate, respectively (Spataro et al., 2019).

Removal efficiencies of phenolic compounds from domestic wastewaters and agricultural runoffs ranged from 33 and 96%, with an average concentration of 5.3 μg/L in the final effluents (Salaudeen et al., 2019). There is contradictory information in the literature with regards to what is the mechanism for phenol degradation in wastewater, where one group claimed biotransformation (Zhong et al., 2010) whereas another study argued that the phenolic compounds are removed mainly through adsorption (Salaudeen et al., 2019). Although activated carbon is the most applied treatment for the removal of phenols it is also very expensive to use (Villegas et al., 2016).

### Peroxygen

Without leaving any by-products, peroxygen decomposes fully into hydrogen and water (Gehr et al., 2009). Due to the high reaction kinetics, there are no longer term traces of peroxygen residue in water bodies. However, some hydrogen peroxide concentrations are detected in natural waters, the formation of which is attributed to photochemical reactions by sunlight (Cooper et al., 1988). In the wastewater, the same fast reaction kinetics is applicable and leave low traces and therefore influent concentrations to wastewater treatment plants are negligible.

### Quaternary ammonium compounds

Quaternary ammonium compounds (QACs) are strong cleaning and disinfecting agents used extensively during the SARS-CoV-2 pandemic. The US Environmental Protection Agency's (USEPA) list of recommended disinfectants against SARS-CoV-2 contains a majority of QAC containing compounds (EPA, 2022), hence Zheng et al. (2020a) found an increased detected level of 19 QACs in household dust. Approximately 75% of QACs used yearly are discharged into wastewater treatment systems (Ismail et al., 2010). QACs concentrations in 12 municipal WWTPs in the Saint Paul Minneapolis urban area ranged between 0.4 and 6.6 μg/L (Pati and Arnold, 2020). Average co- concentrations of QACs ranged between 2 and 72 ng/L and antibiotics (ciprofloxacin, erythromycin, clarithromycin, trimethoprim, metronidazole) ranged between 36 and 129 ng/L were observed in treated effluent of several WWTPs in Sweden (Östman et al., 2017). Widespread detection of Benzalkonium chloride (BAC) in samples obtained from different WWTP effluents demonstrates that biological processes are not the most effective treatment method for QACs (Zhang et al., 2015). The maximum concentration of BACs in 5 WWTPs located in two different Austrian cities were found to be 170 μg/L in the influent, and 4.1 μg/L in the effluent (Kreuzinger et al., 2007). QAC compounds are biodegradable under aerobic conditions; however, their sorption rates are faster than their degradation rates (Zhang et al., 2015). QACs are cationic surfactants and have a strong affinity to anionic surfaces such as biomass (Ferrer and Furlong, 2001; Hajaya and Pavlostathis, 2012). These properties combined with their long half-lives led to the accumulation of QACs on the sewage sludge (Tezel and Pavlostathis, 2009). Although the removal efficiency for QACs in WWTPs is above 90% (Clara et al., 2007; Kreuzinger et al., 2007), only 20% are removed by biotransformation. Therefore, ~70% are adsorbed to the sludge, which will be returned to the environment through land application of QACs-bearing biosolids (Ismail et al., 2010; Zhang et al., 2015). According to the USEPA BACs are toxic to aquatic inhabitants and they recommend against the discharge of BACs into the receiving water bodies. Therefore, the proper treatment of wastewater containing these compounds is important for environmental health (Pereira and Tagkopoulos, 2019).

## Resistance to disinfectants and antiseptics and cross-resistance between antibiotics

The first described resistance to disinfectants happened in 1887 by Kossiakoff in Paris where he described the adaptation to phenol, boric acid and mercuric chloride (Russell, 2004). In the 1940s and 1950s a number of papers were published studying the development of several DAs in gram positive and negative bacteria (Russell, 2004).

### Mechanism of resistance

The resistance mechanisms for disinfectants resemble those of antibiotics resistance. Generally, these mechanisms reduce the overall concentration of microbicides the organism is exposed to or find a way to evade the compound. These mechanisms are (i) efflux pumps, (ii) enzymatic inactivation, (iii) target modification, (iv) changes in the cell surface to reduce permeability or interaction and (v) by-pass of metabolic pathways (Webber et al., 2008; Gnanadhas et al., 2013).

#### Efflux pumps

Efflux pumps are common among bacteria and there are five main protein families documented: ATP-binding cassette (ABC), drug/metabolite transporter (DMT), multidrug and toxic compound extrusion (MATE), major facilitators (MFS) and resistance nodulation cell division (RND) (Borges-Walmsley and Walmsley, 2001; Piddock, 2006; Poole, 2007). The pumps ensure a lower intracellular level of DA, reduce the sensitivity of the cell and can confer resistance if overexpressed (Chuanchuen et al., 2003; Piddock, 2006; Mima et al., 2007). Efflux pump as a resistance mechanism against QAC and triclosan mediated through e.g., qacA and mexCD in the strains *S. aureus, P. aeruginosa, E. coli* and *A. baumanii* is well documented (Tennent et al., 1989; McMurry et al., 1998; Heir et al., 1999; Chuanchuen et al., 2001; Morita et al., 2003; Mima et al., 2007; Rajamohan et al., 2009; Mc Cay et al., 2010). Transcriptome analysis of *E. coli* and *Salmonella enterica* after exposure to triclosan showed higher expression of efflux genes as well as species specific responses (Bailey et al., 2009).

#### Enzymatic inactivation

Enzymatic inactivation renders the harmful substance incapable from doing further damage. Enzymatic inactivation takes place for metallic ions (Cu^2+^, Ag^+^) which are reduced to their non-effective oxidation states (Cloete, 2003). Aldehydes are inactivated by aldehyde dehydrogenase, and peroxygens *via* the inactivation of free radicals by catalases (Kümmerle et al., 1996), superoxide dismutase and alkyl hydroperoxidases (Greenberg et al., 1990).

#### Changes in cell surface and permeability

Changes in Cell surface and permeability are well documented for gram-positive, gram-negative bacteria, spores, and mycobacteria (Manzoor et al., 1999; Denyer et al., 2002; Lambert, 2002; Fraud et al., 2003; Svetlíková et al., 2009; Frenzel et al., 2011; Leggett et al., 2012; Machado et al., 2013). Gram-negative bacteria change their cell permeability through the reduced expression of porins and by changing lipopolysaccharide expression and structure (Denyer et al., 2002; Machado et al., 2013). For mycobacteria and gram-positive bacteria, the resistance comes from changes in the mycoylacyl arabinogalacatan layer as well as change in porin expression (Manzoor et al., 1999; Lambert, 2002; Fraud et al., 2003; Svetlíková et al., 2009; Frenzel et al., 2011). The resistance of spores through cell permeability is well described by Leggett et al. (2012).

#### By-pass metabolic pathways

By-pass metabolic pathways are not well documented as a resistance mechanism for DAs. There are several organisms where the higher resistance could be attributed to changes in metabolism. For *S. enterica*, triclosan resistances was associated with by-pass metabolic pathways and was discovered using proteomics (Webber et al., 2008). As previously mentioned, a target for triclosan is the lipid metabolism *via* the enoyl-acyl carrier protein reductase (FabI) enzyme (Larras et al., 2020). Webber et al. (2008) found nine proteins which were involved in the production of pyruvate and fatty acids with altered expression after exposure to triclosan. In a study by Tkachenko et al. (2007) investigating triclosan resistant *S. aureus*, a change in the lipid composition of the cell membrane was found resulting in a change in the expression profile for branched chain lipid acids.

#### Biofilm

Biofilm formation is a more general ecophysiological defense mechanism against antagonists. Bacteria in biofilms are less vulnerable due to transport limitations, predominantly limited to molecular diffusion, and often form a synergistic community comprised of multiple strains. Microbicides have to penetrate several layers of bacteria and exopolymer substance (EPS) driven by a falling concentration gradient which result in lower disinfectant concentrations at the deeper strata (Chen and Stewart, 1996). Additionally, more resistant strains might further enhance the protection of the biofilm for more susceptible strains (Leriche et al., 2003). As an example, *Listeria monocytogenes* is less susceptible to the popular QAC, BAC, and peracetic acid after biofilm formation (Pang et al., 2019; Ibusquiza et al., 2011). At the same time a disinfectant gradient exists in the biofilm with a sub-MIC stimulating the resistome development. As the diffusive transport limitation also apply for all other molecular and particulater components, specific growth is also reduced which partly counteract the benefit of the sessile growth state (Anderl et al., 2003).

### Acquisition and dissemination of resistance

Besides the obvious acquisition through vertical inheritance, microorganism obtain new resistance mechanisms through horizontal gene transfer (HGT) or mutations (Jury et al., 2010). As mutations occur randomly and are then normally disseminated through vertical inheritance, HGT is of greater concern as this enables genes to make phylogenetic jumps (Pál et al., 2005; Barlow, 2009; Treangen and Rocha, 2011; Huang et al., 2017).

The three main mechanisms are conjugation, transformation and transduction. In conjugation processes a plasmid is transmitted *via* a cellular connection from a donor bacterium to a recipient, called trans-conjugant (Thomas, 2000). Transformation occurs by excreted free suspended extracellular DNA (eDNA) being taken up and incorporated into the intracellular genome by competent bacteria (De Vries and Wackernagel, 2002; Heuer and Smalla, 2007). The last mechanism is transduction which involves bacteriophages as transporting shuttle for DNA fragments which are incorporated into the recipient cells genomes (Cano and Colomé, 1988; Snyder and Champness, 2007; Modi et al., 2013). It can further be divided into generalized transduction where DNA segments are randomly packed with the viral DNA, and specialized transduction where a DNA fragment from the viral vicinity is packed alongside the viral DNA (Canchaya et al., 2003).

While all mechanisms have been described for DA resistance mechanism dissemination, the main focus is on conjugation (Bjorland et al., 2005; Mc Carlie et al., 2020; Tong et al., 2021). Especially well researched are *qac* genes encoding multi efflux pumps, shown to occur on IncP1β-plasmids and class 1 integrons opening a wide host range (Schlüter et al., 2007). In the food industry plasmid mediated QAC resistance genes were documented, and dissemination suggested (Bjorland et al., 2005; Li et al., 2017).

### Co- and cross-resistance

Co-resistance describes the mechanism that transfers various genetic components encoding different resistances at the same time (e.g., plasmid, transposons, integrons) (Cantón and Ruiz-Garbajosa, 2011). These genes are then separately expressed and exercise their resistance. In the case of cross-resistance, a microorganism is resistant to several (unrelated) compounds through the same mechanism [e.g., using the same efflux pump against different antibiotics (Masuda et al., 1995, 1996)].

For alcohol-based disinfectants, reports have claimed no cross-resistance between DAs and antibiotics (Kampf, 2018a,b). This is supported by a recent study of Morante et al. (2021) which found no relation in *Klebsiella pneumoniae* isolates resistant to isopropanol and antibiotic resistances. This is contrary to de Carvalho et al. (2020) who showed that *Mycobacterium vaccae* were more resistant to antibiotics after disinfectant exposure. The strains were exposed for 64 h to ethanol-based hand rubs, causing a change in the fatty acid composition of the cell membrane. The resistance of the adapted strains was compared to non-adapted to the antibiotics levofloxacin, teicoplanin and the efflux pump inhibitors thioridazine and omeprazole. All the strains showed a higher resistance than their non-adapted counterparts indicating a cross-resistance through a change in permeability (de Carvalho et al., 2020). In a study looking at 16 *Mycobacterium chelonae* isolates it was found that 50% of the strains were tolerant against 2% glutaraldehyde. All of the glutaraldehyde tolerant strains showed resistance to at least two classes of antibiotics. The addition of efflux pump inhibitors did not change the MIC of glutaraldehyde or antibiotics indicating an alternative resistance mechanism besides efflux pumps (Nomura et al., 2004). For the DAs triclosan, benzalkonium chloride and chlorhexidine a number of examples for cross-resistance exist. Merchel Piovesan Pereira et al. (2021) exposed 40 *E. coli* strains to sub-inhibitory concentrations of 10 widely used DAs. 17 of these strains exhibited cross-resistance to the antibiotics ampicillin, chloramphenicol or norfloxacin. These results showed that the DAs chlorophene, BAC, chlorhexidine and glutaraldehyde induced cross-resistances. Membrane related mutations were overrepresented, and the majority of the strains showed improved biofilm-forming capacities (Merchel Piovesan Pereira et al., 2021). In an investigation, *E. coli* strains were isolated from pigs, pig carcasses and pork, and were subsequently exposed to the DAs triclosan, BAC and chlorhexidine. Afterwards the strains were tested for eight antibiotics. Triclosan caused cross-resistance to all eight antibiotics while BAC and chlorhexidine caused resistance to at least three of the eight different antibiotics. The presence of the efflux pump inhibitor (EPI) Phenylalanine-arginine β-naphthylamide (PAβN) restored susceptibility to chloramphenicol and trimethoprim, while leaving the strains resistant to the other antibiotics (Puangseree et al., 2021). This indicates that the resistance to chloramphenicol and trimethoprim was caused by the expression of efflux pumps but that the cross-resistance is multifaceted and caused by several factors. Comparable results were shown for six *E. coli* and six non-typhoidal *Salmonella* strains which were exposed to QACs. After a 12-day period, all strains showed increased MIC values for the tested antimicrobials where the highest resistance was seen for ampicillin, tetracycline, ciprofloxacin and chloramphenicol. It was additionally shown that PAβN weakly restores susceptibility, indicating only a weak involvement of efflux pumps (Nhung et al., 2015). Fraud et al. (2008) exposed *P. aeruginosa* to cationic disinfectants from the biguanide and quaternary ammonium chloride classes and showed that expression for the *mexCD-oprJ* operon was induced as well as an improved chlorhexidine resistance. MexCD-oprJ is a multidrug efflux pump responsible for tetracycline and ciprofloxacin resistance (Masuda et al., 1995, 1996). In a research article by Gadea et al. (2017) looking at resistance through exposure to QACs, they exposed 76 previously identified biocide and antibiotic sensitive strains from organic foods to the QACs benzalkonium chloride and hexadecylpyridinium (HDP). The strains were exposed *via* serial inoculation to gradually increased concentrations of these DAs. Afterwards the strains were exposed to a multitude of DAs: didecyldimethylammonium bromide, cetrimide, hexachlorophene, hexadecylpyridinium chloride, chlorhexidine, BAC; and the antibiotics ampicillin, cefotaxime, ceftazidime, ciprofloxacin, sulfamethoxazole/trimethoprim, tetracycline and nalidixic acid. Interestingly, exposure to BAC and HDP caused reduced susceptibility to other DAs, among these; hexachlorophene, triclosan and chlorhexidine. BAC exposed strains showed increased resistance to ampicillin, sulfamethoxazole and cefotaxime and increased membrane rigidity. HDP exposed strains showed a more heterogenous antibiotic resistance profile which also proved strain specific (Gadea et al., 2017). In several independent experiments, *Salmonella enterica* was exposed to popular farm disinfectants for different periods of time. Several strains exhibited reduced susceptibility to antibiotics such as ampicillin, tetracycline and ciprofloxacin and they were associated with higher expression of the efflux pump AcrAB-TolC (Karatzas et al., 2007, 2008; Randall et al., 2007). Nicolae Dopcea et al. (2020) exposed different *Staphylococcus* spp. strains to chlorhexidine and showed that it led to higher MICs for chlorhexidine, but also to a lowered sensitivity to popular antibiotics such as ampicillin, gentamicin and tetracycline.

Inferring from this, some DAs, like QAC, triclosan and chlorhexidine are more likely to induce cross-resistance or reduced sensitivity to antibiotics in specific strains. This might well be due to a research bias for certain DA compounds and their effect on popular researched strains. Due to this possible bias further research in this area is needed.

The cross-resistance mechanisms discussed here are mostly related to more general resistance responses, changes in membrane permeability, efflux pumps (Blanco et al., 2016), and the structural effects of biofilms. In this regard it is not surprising that these mechanisms cause cross-resistance as they all decrease the accessibility of DAs and antibiotics. At the same time recent research has shown that EPI only partially restores susceptibility to acquired antibiotic resistances through DA exposure, strongly indicating that efflux pumps are only part of the resistance and that it is most likely a co-development of several mechanisms as has been proposed for *E. coli* in response to BAC (Jang et al., 2008; Moen et al., 2012). These results indicate that cross-resistance between DA resistance mechanisms and antibiotic resistance mechanisms are the result of the general microbial adaptation to a hostile environment.

## Conclusions and future perspectives

This review focused on the occurrence of DAs in aquatic environments, their resistance mechanisms and their increased cross-resistance to antibiotics. Microorganisms can develop resistance to biocidal agents, when there is a constant selective pressure, and simultaneously it may increase the development rate of antibiotic resistance, hence improve their tolerance to antibiotics. Alcohol-based disinfectants promote this to a lesser degree than compounds like QACs, triclosan and chlorhexidine, which have been flagged due to their potential to promote cross-resistance. Together with the assessment of the occurrence and fate of DAs in wastewater treatment plants, this work indicates that some DAs promote the emergence of cross-resistance to antibiotics and should be given special attention.

Regarding the collateral effect of the SARS-CoV-2 pandemic enhanced the use of DAs and therefore resulting ecological concentrations of DAs in streams entering the WWTPs. Therefore, there is an urgent demand for sustainable control and handling of these micropollutants from wastewaters. Although the development of resistance mechanisms against DAs is not avoidable, measures should be implemented to limit and slow down the development of resistances. In this view, following points are suggested:

- Usage of DAs in concentrations that have no promoting effect on the development of resistance mechanisms in the environment.
- Developing new methods or operational strategies with improved removal efficiency of critical DAs such as QACs, biguanides, and bisphenols in WWTPs as these are proven to promote cross-resistance in bacteria.
- Retrofitting existing WWTP to tertiary treatment unit processes capable of conversion of key DA where economically possible.
- Implementing a surveillance system for disinfectant resistant genes in WWTP.

These approaches should be followed and implemented until obtaining a better knowledge about mechanisms of the emergence of DA resistance and understanding how these mechanisms would influence the mechanisms of antibiotic resistance. While progress in this was done in recent years, research must be intensified to evaluate the potential danger level and potential solutions.

## Author contributions

DB researched and wrote the manuscript with special focus on Sections Disinfectants and antiseptics and their mechanism of action and Resistance to disinfectants and antiseptics and cross-resistance between antibiotics. NEH researched and wrote the manuscript with special focus on Sections Disinfectants and antiseptics and their mechanism of action and Occurrence of disinfectants and antiseptics in aquatic environments, and their fate in wastewater treatment plants. CU, KMK, and IP-O researched, wrote, and edited the manuscript. RK provided significant input on wastewater treatment and operation of WWTPs and edited the manuscript. All authors reviewed the manuscript. All authors contributed to the article and approved the submitted version.

## References

[B1] Abbott T. Kor-Bicakci G. Islam M. S. Eskicioglu C. A. (2020). review on the fate of legacy and alternative antimicrobials and their metabolites during wastewater and sludge treatment. Int. J. Mol. Sci. 21, 1–52. 10.3390/ijms21239241 33287448 PMC7729486

[B2] Agency USEP (1993). EPA R.E.D. Facts. Peroxy Compounds. US Environmental Protection Agency.

[B3] Alfa M. J. Sitter D. L. (1994). In-hospital evaluation of orthophthalaldehyde as a high level disinfectant for flexible endoscopes. J. Hosp. Infect. 26, 15–26. 10.1016/0195-6701(94)90075-2 7910179 PMC7124318

[B4] Alfhili M. A. Lee M. H. (2019). Triclosan: An update on biochemical and molecular mechanisms. Oxid. Med. Cell. Longev. 2019. 10.1155/2019/1607304 31191794 PMC6525925

[B5] Ali A. Arshad M. Zahir Z. A. Jamil A. (2011). Influence of triclosan and triclocarban antimicrobial agents on the microbial activity in three physicochemically differing soils of south Australia. Soil Environ. 30, 95–103.

[B6] Allen G. C. Flores-Vergara M. A. Krasynanski S. Kumar S. Thompson W. F. A. (2006). modified protocol for rapid DNA isolation from plant tissues using cetyltrimethylammonium bromide. Nat. Protoc. 1, 2320–2325. 10.1038/nprot.2006.384 17406474

[B7] Allen M. J. Morby A. P. White G. F. (2004). Cooperativity in the binding of the cationic biocide polyhexamethylene biguanide to nucleic acids. Biochem. Biophys. Res. Commun. 318, 397–404. 10.1016/j.bbrc.2004.04.043 15120614

[B8] Allen M. J. White G. F. Morby A. P. (2006). The response of *Escherichia coli* to exposure to the biocide polyhexamethylene biguanide. Microbiology. 152, 989–1000. 10.1099/mic.0.28643-0 16549663

[B9] Allmyr M. Harden F. Toms L. M. L. Mueller J. F. McLachlan M. S. Adolfsson-Erici M.. (2008). The influence of age and gender on triclosan concentrations in Australian human blood serum. Sci. Total Environ. 393, 162–167. 10.1016/j.scitotenv.2007.12.006 18207219

[B10] Anderl J. N. Zahller J. Roe F. Stewart P. S. (2003). Role of nutrient limitation and stationary-phase existence in Klebsiella pneumoniae biofilm resistance to ampicillin and ciprofloxacin. Antimicrob. Agents Chemother. 47, 1251–1256. 10.1128/AAC.47.4.1251-1256.2003 12654654 PMC152508

[B11] Armstrong D. L. Lozano N. Rice C. P. Ramirez M. Torrents A. (2018). Degradation of triclosan and triclocarban and formation of transformation products in activated sludge using benchtop bioreactors. Environ. Res. 161, 17–25. 10.1016/j.envres.2017.10.048 29096316

[B12] Bailey A. M. Constantinidou C. Ivens A. Garvey M. I. Webber M. A. Coldham N.. (2009). Exposure of *Escherichia coli* and *Salmonella enterica* serovar Typhimurium to triclosan induces a species-specific response, including drug detoxification. J. Antimicrob. Chemother. 64, 973–985. 10.1093/jac/dkp320 19759044

[B13] Baldry M. G. C. (1983). The bactericidal, fungicidal and spicidal properties of hydrogen peroxide and peracetic acid. Bac. 54, 417–23. 10.1111/j.1365-2672.1983.tb02637.x 6409877

[B14] Barancheshme F. Munir M. (2018). Strategies to combat antibiotic resistance in the wastewater treatment plants. Front. Microbiol. 8, 2603. 10.3389/fmicb.2017.02603 29387043 PMC5776126

[B15] Barlow M. (2009). What Antimicrobial Resistance Has Taught Us About Horizontal Gene Transfer. Horiz. gene Transf. Methods. Mol Biol. 532, 397–411. 10.1007/978-1-60327-853-9_23 19271198

[B16] Beeuwkes H. (1958). The use of chlorhexidine. Antonie van Leeuwenhoek 24, 49–62. 10.1007/BF02548431 13595630

[B17] Bellat J. P. Bezverkhyy I. Weber G. Royer S. Averlant R. Giraudon J. M.. (2015). Capture of formaldehyde by adsorption on nanoporous materials. J. Hazard. Mater. 300, 711–717. 10.1016/j.jhazmat.2015.07.078 26296074

[B18] Bennett J. E. Dolin R. Blaser M. J. (2015). Mandell, Douglas, and Bennett's Principles and Practice of Infectious Diseases. Philadelphia, PA: Elsevier.

[B19] Bester K. (2005). Fate of triclosan and triclosan-methyl in sewage treatment plants and surface waters. Arch. Environ. Contam. Toxicol. 49, 9–17. 10.1007/s00244-004-0155-4 15959704

[B20] Beveridge C. M. Parr A. C. S. Smith M. J. Kerr A. Cowling M. J. Hodgkiess T.. (1998). The effect of benzalkonium chloride concentration on nine species of marine diatom. Environ. Pollut. 103, 31–36. 10.1016/S0269-7491(98)00134-1

[B21] Bhargava H. N. Leonard P. A. (1996). Triclosan: applications and safety. Am. J. Infect. Control. 24, 209–218. 10.1016/S0196-6553(96)90017-6 8807001

[B22] Bjorland J. Steinum T. Kvitle B. Waage S. Sunde M. Heir E.. (2005). Widespread distribution of disinfectant resistance genes among staphylococci of bovine and caprine origin in Norway. J. Clin. Microbiol. 43, 4363–4368. 10.1128/JCM.43.9.4363-4368.2005 16145078 PMC1234083

[B23] Blanco P. Hernando-Amado S. Reales-Calderon J. A. Corona F. Lira F. Alcalde-Rico M.. (2016). Bacterial multidrug efflux pumps: Much more than antibiotic resistance determinants. Microorganisms. 4, 1–19. 10.3390/microorganisms4010014 27681908 PMC5029519

[B24] Bloomfield S. F. (1996). Chlorine and iodine formulations, in Handbook of Disinfectants and Antiseptics. New York City, NY: CRC Press. p. 147–53.

[B25] Boillot C. Perrodin Y. (2008). Joint-action ecotoxicity of binary mixtures of glutaraldehyde and surfactants used in hospitals: use of the Toxicity Index model and isoblogram representation. Ecotoxicol. Environ. Saf. 71, 252–259. 10.1016/j.ecoenv.2007.08.010 17945345

[B26] Boleda M. R. Galceran M. T. Ventura F. (2011). Behavior of pharmaceuticals and drugs of abuse in a drinking water treatment plant (DWTP) using combined conventional and ultrafiltration and reverse osmosis (UF/RO) treatments. Environ. Pollut. 159, 1584–91. 10.1016/j.envpol.2011.02.051 21459501

[B27] Bolz U. Hagenmaier H. Ko W. (2001). Phenolic xenoestrogens in surface water, sediments, and sewage sludge from Baden-Wurttemberg. Environ. Pollut. 115, 291–301. 10.1016/S0269-7491(01)00100-2 11706802

[B28] Bonesvoll P. Gjermo P. (1978). A comparison between chlorhexidine and some quaternary ammonium compounds with regard to retention, salivary concentration and plaque-inhibiting effect in the human mouth after mouth rinses. Arch. Oral Biol. 23, 289–294. 10.1016/0003-9969(78)90021-3 278566

[B29] Borges-Walmsley M. I. Walmsley A. R. (2001). The structure and function of drug pumps. Trends Microbiol. 9, 71–79. 10.1016/S0966-842X(00)01920-X 11173246

[B30] Borkow G. Gabbay J. (2005). Copper as a biocidal tool. Curr. Med. Chem. 12, 2163–2175. 10.2174/0929867054637617 16101497

[B31] Bowes J. H. Cater C. W. (1968). The interaction of aldehydes with collagen. BBA - Protein Struct. 168, 341–352. 10.1016/0005-2795(68)90156-6 5748675

[B32] Boyce J. M. (2018). Alcohols as surface disinfectants in healthcare settings. Infect. Control Hosp. Epidemiol. 39, 323–328. 10.1017/ice.2017.301 29374503

[B33] Brient J. A. Manning M. J. Freeman M. H. (2020). Copper naphthenate - protecting America's infrastructure for over 100 years and its potential for expanded use in Canada and Europe. Wood Mater. Sci. Eng. 15, 368–376. 10.1080/17480272.2020.1837948

[B34] Bruch M. K. (1996). Chloroxylenol: An Old-New antimicrobial, in Handbook of Disinfectants and Antiseptics. New York City, NY: CRC Press.

[B35] Bryant C. W. Avenell J. J. Barkley W. A. Thut R. N. (1992). The removal of chlorinated organics from conventional pulp and paper wastewater treatment systems. Water Sci. Technol. 26, 417–425. 10.2166/wst.1992.0421

[B36] Bush L. W. Benson L. M. White J. H. (1986). Pig skin as test substrate for evaluating topical antimicrobial activity. J. Clin. Microbiol. 24, 343–348. 10.1128/jcm.24.3.343-348.1986 3093525 PMC268910

[B37] Canchaya C. Fournous G. Chibani-Chennoufi S. Dillmann M. L. Brüssow H. (2003). Phage as agents of lateral gene transfer. Curr. Opin. Microbiol. 6, 417–424. 10.1016/S1369-5274(03)00086-9 12941415

[B38] Cano R. J. Colomé J. S. (1988). Essentials of microbiology, in Thomson Learning. Boston, MA: Thomson Learning. p. 649.

[B39] Cantón R. Ruiz-Garbajosa P. (2011). Co-resistance: An opportunity for the bacteria and resistance genes. Curr. Opin. Pharmacol. 11, 477–485. 10.1016/j.coph.2011.07.007 21840259

[B40] Capkin E. Ozcelep T. Kayis S. Altinok I. (2017). Antimicrobial agents, triclosan, chloroxylenol, methylisothiazolinone and borax, used in cleaning had genotoxic and histopathologic effects on rainbow trout. Chemosphere. 182, 720–9. 10.1016/j.chemosphere.2017.05.093 28531838

[B41] Carey D. E. McNamara P. J. (2014). The impact of triclosan on the spread of antibiotic resistance in the environment. Front. Microbiol. 5, 1–11. 10.3389/fmicb.2014.00780 25642217 PMC4295542

[B42] Carletti G. Fatone F. Bolzonella D. Cecchi F. (2008). Occurrence and fate of heavy metals in large wastewater treatment plants treating municipal and industrial wastewaters. Water Sci. Technol. 57, 1329–1336. 10.2166/wst.2008.230 18495995

[B43] Catherine H. Penninckx M. Frédéric D. (2016). Product formation from phenolic compounds removal by laccases: a review. Environ. Technol. Innov. 5, 250–266. 10.1016/j.eti.2016.04.001

[B44] Cervantes-Avilés P. Huang Y. Keller A. A. (2019). Incidence and persistence of silver nanoparticles throughout the wastewater treatment process. Water Res. 156, 188–198. 10.1016/j.watres.2019.03.031 30913422

[B45] Chappell J. B. Greville G. D. (1954). Effect of Silver Ions on mitochonrial adenosine triphosphate. Nature. 174, 930–931. 10.1038/174930b0 13214046

[B46] Chawner J. A. Gilbert P. (1989a). Interaction of the bisbiguanides chlorhexidine and alexidine with phospholipid vesicles: evidence for separate modes of action. J. Appl. Bacteriol. 66, 253–258. 10.1111/j.1365-2672.1989.tb02476.x 2663802

[B47] Chawner J. A. Gilbert P. A. (1989b). comparative study of the bactericidal and growth inhibitory activities of the bisbiguanides alexidine and chlorhexidine. J. Appl. Bacteriol. 66, 243–252. 10.1111/j.1365-2672.1989.tb02475.x 2663801

[B48] Chen X. Stewart P. S. (1996). Chlorine penetration into artificial biofilm is limited by a reaction-diffusion interaction. Environ. Sci. Technol. 30, 2078–2083. 10.1021/es9509184

[B49] Chhetri R. K. Baun A. Andersen H. R. (2019). Acute toxicity and risk evaluation of the CSO disinfectants performic acid, peracetic acid, chlorine dioxide and their by-products hydrogen peroxide and chlorite. Sci. Total Environ. 677, 1–8. 10.1016/j.scitotenv.2019.04.350 31051379

[B50] Chindera K. Mahato M. Kumar Sharma A. Horsley H. Kloc-Muniak K. Kamaruzzaman N. F.. (2016). The antimicrobial polymer PHMB enters cells and selectively condenses bacterial chromosomes. Sci. Rep. 6, 1–13. 10.1038/srep23121 26996206 PMC4800398

[B51] Choi D. Oh S. (2019). Removal of chloroxylenol disinfectant by an activated sludge microbial community. Microbes Environ. 34, 129–135. 10.1264/jsme2.ME18124 30799319 PMC6594741

[B52] Chuanchuen R. Beinlich K. Hoang T. T. Becher A. Karkhoff-Schweizer R. R. Schweizer H. P.. (2001). Cross-resistance between triclosan and antibiotics in *Pseudomonas aeruginosa* is mediated by multidrug efflux pumps: Exposure of a susceptible mutant strain to triclosan selects nfxB mutants overexpressing MexCD-OprJ. Antimicrob. Agents Chemother. 45, 428–432. 10.1128/AAC.45.2.428-432.2001 11158736 PMC90308

[B53] Chuanchuen R. Karkhoff-Schweizer R. A. R. Schweizer H. P. (2003). High-level triclosan resistance in *Pseudomonas aeruginosa* is solely a result of efflux. Am. J. Infect. Control. 31, 124–127. 10.1067/mic.2003.11 12665747

[B54] Clara M. Scharf S. Scheffknecht C. Gans O. (2007). Occurrence of selected surfactants in untreated and treated sewage. Water Res. 41, 4339–4348. 10.1016/j.watres.2007.06.027 17624392

[B55] Clara M. Windhofer G. Weilgony P. Gans O. Denner M. Chovanec A.. (2012). Identification of relevant micropollutants in Austrian municipal wastewater and their behaviour during wastewater treatment. Chemosphere. 87, 1265–72. 10.1016/j.chemosphere.2012.01.033 22342340

[B56] Cloete T. E. (2003). Resistance mechanisms of bacteria to antimicrobial compounds. Int. Biodeterior. Biodegrad. 51, 277–282. 10.1016/S0964-8305(03)00042-8

[B57] Collignon P. (2017). Fact sheets on sustainable development goals: health targets: Antimicrobial resistance. Emerging Infect. Dis. 6, 434–436. 10.3201/eid0604.000428 10905989 PMC2640893

[B58] Cooper W. J. Zika R. G. Petasne R. G. Plane J. M. C. (1988). Photochemical formation of H2O2 in natural waters exposed to sunlight. Environ. Sci. Technol. 22, 1156–1160. 10.1021/es00175a004 22148607

[B59] Cossa D. Fileman C. (1991). Mercury concentrations in surface waters of the English channel: a cooperative study. Mar. Pollut. Bull. 22, 197–200. 10.1016/0025-326X(91)90470-D

[B60] Coulthard C. E. Sykes G. (1936). The germicidal effect of alcohol with special reference to its action on bacterial spores. Pharm. J. 137, 79–81.

[B61] Cui Y. Shi X. Guang C. Zhang Z. Wang C. Wang C.. (2019). Comparison of pressure-swing distillation and heterogeneous azeotropic distillation for recovering benzene and isopropanol from wastewater. Process Saf. Environ. Prot. 122, 1–12. 10.1016/j.psep.2018.11.017

[B62] Davidson P. M. Branden A. L. (1981). Antimicrobial activity of non-halogenated phenolic compounds. J. Food Prot. 44, 623–632. 10.4315/0362-028X-44.8.623 30836539

[B63] Davies A. Bentley M. Field B. S. (1968). Comparison of the action of vantocil, cetrimide and chlorhexidine on *Escherichia coli* and its spheroplasts and the protoplasts of gram positive bacteria. J. Appl. Bacteriol. 31, 448–461. 10.1111/j.1365-2672.1968.tb00394.x 4973618

[B64] Davies G. E. Francis J. Martin A. R. Rose F. L. Swain G. (1954). 1:6-Di-4'-chlorophenyldiguanidohexane (hibitane); laboratory investigation of a new antibacterial agent of high potency. Br. J. Pharmacol. Chemother. 9, 192–196. 10.1111/j.1476-5381.1954.tb00840.x 13172429 PMC1509439

[B65] De Almeida J. Hoogenkamp M. Felippe W. T. Crielaard W. Van Der Waal S. V. (2016). Effectiveness of EDTA and modified salt solution to detach and kill cells from enterococcus faecalis biofilm. J. Endod. 42, 320–3. 10.1016/j.joen.2015.11.017 26723483

[B66] de Carvalho C. C. C. R. Teixeira R. Fernandes P. (2020). Mycobacterium vaccae adaptation to disinfectants and hand sanitisers, and evaluation of cross-tolerance with antimicrobials. Antibiotics. 9, 1–16. 10.3390/antibiotics9090544 32867093 PMC7559525

[B67] de León L. López M. R. Moujir L. (2010). Antibacterial properties of zeylasterone, a triterpenoid isolated from Maytenus blepharodes, against *Staphylococcus aureus*. Microbiol. Res. 165, 617–626. 10.1016/j.micres.2009.12.004 20116223

[B68] De Vries J. Wackernagel W. (2002). Integration of foreign DNA during natural transformation of Acinetobacter sp. by homology-facilitated illegitimate recombination. Proc. Natl. Acad. Sci. USA. 99, 2094–2099. 10.1073/pnas.042263399 11854504 PMC122324

[B69] Denyer S. P. Hodges N. A. Gorman S. P. Denyer S. Russell A. D. (2004). Chapter 18 Non-Antibiotic Antibacterial Agents : Mode of Action and Resistance. Malden, MA: Wiley-Blackwell Science.

[B70] Denyer S. P. Maillard J. -Y. (2002). Cellular impermeability and uptake of biocides and antibiotics in Gram-positive bacteria and mycobacteria. J Appl Microbiol Symp Suppl. 92, 35–45. 10.1046/j.1365-2672.92.5s1.19.x 12000612

[B71] Denyer S. P. Stewart G. S. A. B. (1998). Mechanisms of action of disinfectants. Int Biodeterior Biodegrad. 41, 261–268. 10.1016/S0964-8305(98)00023-7

[B72] Dettenkofer M. Spencer R. C. (2007). Importance of environmental decontamination - a critical view. J. Hosp. Infect. 65, 55–57. 10.1016/S0195-6701(07)60016-4 17540243

[B73] Dibrov P. Dzioba J. Gosink K. K. Häse C. C. (2002). Chemiosmotic mechanism of antimicrobial activity of Ag+ in Vibrio cholerae. Antimicrob. Agents Chemother. 46, 2668–2670. 10.1128/AAC.46.8.2668-2670.2002 12121953 PMC127333

[B74] Dizdaroglu M. Jaruga P. (2012). Mechanisms of free radical-induced damage to DNA. Free Radic. Res. 46, 382–419. 10.3109/10715762.2011.653969 22276778

[B75] Dizman B. Elasri M. O. Mathias L. J. (2004). Synthesis and antimicrobial activities of new water-soluble bis-quaternary ammonium methacrylate polymers. J. Appl. Polym. Sci. 94, 635–642. 10.1002/app.20872

[B76] Drewes J. E. Fox P. Jekel M. (2001). Occurrence of iodinated X-ray contrast media in domestic effluents and their fate during indirect potable reuse. J. Environ. Sci. Heal - Part A Toxic/Hazardous Subst. Environ. Eng. 36, 1633–1645. 10.1081/ESE-100106248 11688680

[B77] Dsikowitzky L. Sträter M. Dwiyitno A. F Irianto H. E. Schwarzbauer J. (2016). First comprehensive screening of lipophilic organic contaminants in surface waters of the megacity Jakarta, Indonesia. Mar. Pollut. Bull. 110, 654–64. 10.1016/j.marpolbul.2016.02.019 26880129

[B78] Dyer C. Hutt L. P. Burky R. Joshi L. T. (2019). Biocide resistance and transmission of Clostridium difficile spores spiked onto clinical surfaces from an American health care facility. Appl. Environ. Microbiol. 85, e01090-19. 10.1128/AEM.01090-19 31300397 PMC6696958

[B79] ECHA (2017). Biocidal Products Committee (BPC) Opinion. Opinion of the Biocidal Products Committee on the application for approval of the active substance PHMB (1600;1,8). ECHA/BPC/059/2015 adopted. p. 1–12. Available online at: https://echa.europa.eu/documents/10162/1167923/phmb_pt02_final_opinion_en.pdf/268e4aac-51c8-4b6f-bf12-e628bdb61677 (accessed November 28, 2022).

[B80] Edwards F. G. Egemen E. Brennan R. Nirmalakhandan N. (1999). Ranking of toxics release: inventory chemicals using a level III fugacity model and toxicity. Water Sci. Technol. 39, 83–90. 10.1016/S0273-1223(99)00257-7

[B81] Eiroa M. Kennes C. Veiga M. C. (2005). Simultaneous nitrification and formaldehyde biodegradation in an activated sludge unit. Bioresour. Technol. 96, 1914–1918. 10.1016/j.biortech.2005.01.041 16084371

[B82] Elekhnawy E. Sonbol F. Abdelaziz A. Elbanna T. (2020). Potential impact of biocide adaptation on selection of antibiotic resistance in bacterial isolates. Futur J. Pharm. Sci. 6, 97. 10.1186/s43094-020-00119-w33674437

[B83] Emmanuel E. Keck G. Blanchard J. M. Vermande P. Perrodin Y. (2004). Toxicological effects of disinfections using sodium hypochlorite on aquatic organisms and its contribution to AOX formation in hospital wastewater. Environ. Int. 30, 891–900. 10.1016/j.envint.2004.02.004 15196837

[B84] EPA (2022). Disinfectants for Coronavirus (COVID-19). Available online at: https://www.epa.gov/coronavirus/list-n-advanced-search-page-disinfectants-coronavirus-covid-19 (accessed November 28, 2022).

[B85] EU (2015). Evaluation of active substances. assessment report. hydrogen peroxide product-types 1-6. Report, Assess. (2015). 1–88. Available online at: https://echa.europa.eu/documents/10162/3547327/7989_1315-01_Assessment_Report.pdf/f4b6ac51-c4e8-b45c-f7ba-b38f48f3cf67 (accessed November 28, 2022).

[B86] European Commission (2016). Commision Implementing Regulation (EU) 2016/672 of 29 April 2016 approving peracetic acid as an existing active substance for use in biocidal products for product-types. p. 48–119. Available online at: https://eur-lex.europa.eu/legal-content/EN/TXT/?uri=CELEX:32016R0672 (accessed November 28, 2022).

[B87] European Committee for Standardization (2020). Chemical Disinfectants and Antiseptics - Application of European Standards for Chemical Disinfectants and Antiseptics. Available online at: https://www.sls.se/globalassets/sls/sls/remissvar/remisser/pren-14885_41_e_stf.pdf (accessed November 28, 2022).

[B88] Fang J. L. Stingley R. L. Beland F. A. Harrouk W. Lumpkins D. L. Howard P.. (2010). Occurrence, efficacy, metabolism, and toxicity of triclosan. J. Environ. Sci. Heal. - Part C Environ. Carcinog. Ecotoxicol. Rev. 28, 147–171. 10.1080/10590501.2010.504978 20859822

[B89] Feng Q. L. Wu J. Chen G. Q. Cui F. Z. Kim T. N. Kim J. O.. (2000). A mechanistic study of the antibacterial effect of silver ions on *Escherichia coli* and *Staphylococcus aureus*. J. Biomed. Mater. Res. 52, 662–668. 10.1002/1097-4636(20001215)52:4<662::aid-jbm10>3.0.co;2-3 11033548

[B90] Ferreira Guedes S. Leitão A. L. (2012). Effect of phenolic compounds and osmotic stress on the expression of penicillin biosynthetic genes from Penicillium chrysogenum var. halophenolicum strain. J. Xenobiotics. 2, 2. 10.4081/xeno.2012.e2

[B91] Ferrer I. Furlong E. T. (2001). Identification of alkyl dimethylbenzylammonium surfactants in water samples by solid-phase extraction followed by ion trap LC/MS and LC/MS/MS. Environ. Sci. Technol. 35, 2583–2588. 10.1021/es001742v 11432568

[B92] Finnegan S. Percival S. L. (2015). EDTA: An antimicrobial and antibiofilm agent for use in wound care. Adv. Wound Care. 4, 415–421. 10.1089/wound.2014.0577 26155384 PMC4486448

[B93] Fraud S. Campigotto A. J. Chen Z. Poole K. (2008). MexCD-OprJ multidrug efflux system of *Pseudomonas aeruginosa*: Involvement in chlorhexidine resistance and induction by membrane-damaging agents dependent upon the AlgU stress response sigma factor. Antimicrob. Agents Chemother. 52, 4478–4482. 10.1128/AAC.01072-08 18838593 PMC2592872

[B94] Fraud S. Hann A. C. Maillard J. Y. Russell A. D. (2003). Effects of ortho-phthalaldehyde, glutaraldehyde and chlorhexidine diacetate on Mycobacterium chelonae and Mycobacterium abscessus strains with modified permeability. J. Antimicrob. Chemother. 51, 575–584. 10.1093/jac/dkg099 12615857

[B95] Fraud S. Maillard J. Y. Russell A. D. (2001). Comparison of the mycobactericidal activity of ortho-phthalaldehyde, glutaraldehyde and other dialdehydes by a quantitattive suspension test. J. Hosp. Infect. 48, 214–221. 10.1053/jhin.2001.1009 11439009

[B96] Frenzel E. Schmidt S. Niederweis M. Steinhauer K. (2011). Importance of porins for biocide efficacy against Mycobacterium smegmatis. Appl. Environ. Microbiol. 77, 3068–3073. 10.1128/AEM.02492-10 21398489 PMC3126410

[B97] Gadea R. Fernández Fuentes M. Á. Pérez Pulido R. Gálvez A. Ortega E. (2017). Effects of exposure to quaternary-ammonium-based biocides on antimicrobial susceptibility and tolerance to physical stresses in bacteria from organic foods. Food Microbiol. 63, 58–71. 10.1016/j.fm.2016.10.037 28040182

[B98] Gardner M. Jones V. Comber S. Scrimshaw M. D. Coello-Garcia T. Cartmell E.. (2013). Performance of UK wastewater treatment works with respect to trace contaminants. Sci. Total Environ. (2013) 456–57, 359–69. 10.1016/j.scitotenv.2013.03.088 23624009

[B99] Gehr R. Chen D. Moreau M. (2009). Performic acid (PFA): Tests on an advanced primary effluent show promising disinfection performance. Water Sci. Technol. 59, 89–96. 10.2166/wst.2009.761 19151490

[B100] Geier D. A. King P. G. Hooker B. S. Dórea J. G. Kern J. K. Sykes L. K.. (2015). Thimerosal: clinical, epidemiologic and biochemical studies. Clin. Chim. Acta. 444, 212–20. 10.1016/j.cca.2015.02.030 25708367

[B101] Giger W. Schaffner C. Kohler H. P. E. (2006). Benzotriazole and tolyltriazole as aquatic contaminants. 1. Input and occurrence in rivers and lakes. Environ. Sci. Technol. 40, 7186–7192. 10.1021/es061565j 17180965

[B102] Gilbert P. Moore L. E. (2005). Cationic antiseptics: diversity of action under a common epithet. J. Appl. Microbiol. 99, 703–715. 10.1111/j.1365-2672.2005.02664.x 16162221

[B103] Gilpin I. M. F. Ullrich M. Wünsche T. Zarschler K. Lebeda O. Pietzsch J.. (2021). Radiolabelled cyclic bisarylmercury: high chemical and in vivo stability for theranostics. Chem. Med. Chem. 16, 2645–2649. 10.1002/cmdc.202100131 33949125 PMC8518081

[B104] Gjemro P. Rolla G. Arskaug L. (1973). Effect on dental plaque formation and some in vitro properties of 12 bis-biguanides. 10.1111/j.1600-0765.1973.tb02169.x 4269606

[B105] Gnanadhas D. P. Marathe S. A. Chakravortty D. (2013). Biocides-resistance, cross-resistance mechanisms and assessment. Expert Opin. Investig. Drugs. 22, 1–16. 10.1517/13543784.2013.748035 23215733

[B106] Gomes B. P. F. A. Ferraz C. C. R. Vianna M. E. Berber V. B. Teixeira F. B. Souza-Filho F. J.. (2001). In vitro antimicrobial activity of several concentrations of sodium hypochlorite and chlorhexidine gluconate in the elimination of Enterococcus faecalis. Int. Endod. J. 34, 424–428. 10.1046/j.1365-2591.2001.00410.x 11556507

[B107] Gong T. Zhang X. (2013). Determination of iodide, iodate and organo-iodine in waters with a new total organic iodine measurement approach. Water Res. 47, 6660–9. 10.1016/j.watres.2013.08.039 24075720

[B108] Gorman S. P. Scott E. M. Russell A. D. (1980). Antimicrobial activity, uses and mechanism of action of glutaraldehyde. J. Appl. Bacteriol. 48, 161–190. 10.1111/j.1365-2672.1980.tb01217.x 6780502

[B109] Graham R. Barger E. H. (1936). Studies on incubator hygiene IV, A note on the virucidal effect of formaldehyde on fowl pox virus. Poult. Sci. 15, 48–52. 10.3382/ps.0150048

[B110] Greenberg J. T. Monach P. Chou J. H. Josephy P. D. Demple B. (1990). Positive control of a global antioxidant defense regulon activated by superoxide-generating agents in *Escherichia coli*. Proc. Natl. Acad. Sci. USA. 87, 6181–6185. 10.1073/pnas.87.16.6181 1696718 PMC54496

[B111] Gregory A. W. Schaalje G. B. Smart J. D. Richard A. (1999). The Mycobactericidal Efficacy of ortho-Phthalaldehyde and the comperative resistances. Infect. Control Hosp. Epidemiol. 20, 324–330. 10.1086/501625 10349948

[B112] Guerra P. Teslic S. Shah A. Albert A. Gewurtz S. B. Smyth S. A.. (2019). Occurrence and removal of triclosan in Canadian wastewater systems. Environ. Sci. Pollut. Res. 26, 31873–31886. 10.1007/s11356-019-06338-w 31489545

[B113] Gulyas H. von Bismarck R. Hemmerling L. (1995). Treatment of industrial wastewaters with ozone/hydrogen peroxide. Water Sci. Technol. 32, 127–34. 10.1016/0273-1223(96)00056-X15318503

[B114] Hajaya M. G. Pavlostathis S. G. (2012). Fate and effect of benzalkonium chlorides in a continuous-flow biological nitrogen removal system treating poultry processing wastewater. Bioresour. Technol. 118, 73–81. 10.1016/j.biortech.2012.05.050 22705509

[B115] Halden R. U. (2014). On the need and speed of regulating triclosan and triclocarban in the United States. Environ. Sci. Technol. 48, 3603–3611. 10.1021/es500495p 24588513 PMC3974611

[B116] Halden R. U. Paull D. H. (2005). Co-occurrence of triclocarban and triclosan in U.S. water resources. Environ Sci Technol. 39, 1420–1426. 10.1021/es049071e 15819193

[B117] Hamilton W. A. (1970). Membrane active antibacterial compounds. Proc Biochem Soc. 70, 118. 10.1042/bj1180046P 4990589 PMC1179229

[B118] Han J. Won E. J. Hwang U. K. Kim I. C. Yim J. H. Lee J. S.. (2016). Triclosan (TCS) and Triclocarban (TCC) cause lifespan reduction and reproductive impairment through oxidative stress-mediated expression of the defensome in the monogonont rotifer (Brachionus koreanus). Comp. Biochem. Physiol. Part - C Toxicol. Pharmacol. (2016) 185–186, 131–7. 10.1016/j.cbpc.2016.04.002 27067728

[B119] Haque H. Russell A. D. (1974). Effect of chelating agents on the susceptibility of some strains of gram-negative bacteria to some antibacterial agents. Antimicrob. Agents Chemother. 6, 200–206. 10.1128/AAC.6.2.200 15828192 PMC444627

[B120] Hargreaves A. J. Constantino C. Dotro G. Cartmell E. Campo P. (2018). Fate and removal of metals in municipal wastewater treatment: a review. Environ. Technol. Rev. 7, 1–18. 10.1080/21622515.2017.1423398

[B121] Heidler J. Halden R. U. (2009). Fate of organohalogens in US wastewater treatment plants and estimated chemical releases to soils nationwide from biosolids recycling. J. Environ. Monit. 11, 2207–2215. 10.1039/b914324f 20024018 PMC2802102

[B122] Heidler J. Sapkota A. Halden R. U. (2006). Partitioning, persistence, and accumulation in digested sludge of the topical antiseptic triclocarban during wastewater treatment. Environ. Sci. Technol. 40, 3634–3639. 10.1021/es052245n 16786704 PMC2768036

[B123] Heir E. Sundheim G. Holck A. L. (1999). The qacG gene on plasmid pST94 confers resistance to quaternary ammonium compounds in staphylococci isolated from the food industry. J. Appl. Microbiol. 86, 378–388. 10.1046/j.1365-2672.1999.00672.x 10196743

[B124] Heuer H. Smalla K. (2007). Horizontal gene transfer between bacteria. Environ. Biosafety Res. 4, 3–13. 10.1051/ebr:2007034 17961477

[B125] Hidalgo A. Lopategi A. Prieto M. Serra J. L. Llama M. J. (2002). Formaldehyde removal in synthetic and industrial wastewater by Rhodococcus erythropolis UPV-1. Appl. Microbiol. Biotechnol. 58, 260–263. 10.1007/s00253-001-0876-5 11876421

[B126] Holloway P. M. Bucknall R. A. Denton G. W. (1986). The effects of sub-lethal concentrations of chlorhexidine on bacterial pathogenicity. J. Hosp. Infect. 8, 39–46. 10.1016/0195-6701(86)90103-9 2875100

[B127] Hopwood D. Allen C. R. McCabe M. (1970). The reactions between glutaraldehyde and various proteins. An investigation of their kinetics. Histochem. J. 2, 137–150. 10.1007/BF01003541 5525781

[B128] Huang W. Tsai L. Li Y. Hua N. Sun C. Wei C.. (2017). Widespread of horizontal gene transfer in the human genome. BMC Genomics. 18, 1–11. 10.1186/s12864-017-3649-y 28376762 PMC5379729

[B129] Hugo W. B. (1992). Disinfection Mechanisms. Principles and Practice of Disinfection, Preservation and Sterilization. (1992) p. 187–210.

[B130] Hugo W. B. Longworth A. R. (1965). Cytological aspects of the mode of action of chlorhexidine diacetate. J. Pharm. Pharmacol. 17, 28–32. 10.1111/j.2042-7158.1965.tb07562.x 14285692

[B131] Ibusquiza P. S. Herrera J. J. R. Cabo M. L. (2011). Resistance to benzalkonium chloride, peracetic acid and nisin during formation of mature biofilms by Listeria monocytogenes. Food Microbiol. 28, 418–25. 10.1016/j.fm.2010.09.014 21356446

[B132] Information NC for B (2004). PubChem Compound Summary for CID 7547, Triclocarban. Available online at: https://pubchem.ncbi.nlm.nih.gov/compound/Triclocarban (accessed November 13, 2022)

[B133] Ingram L. O. N. Buttke T. M. (1985). Effects of alcohols on micro-organisms. Adv. Microb. Physiol. 25, 253–300. 10.1016/S0065-2911(08)60294-5 6398622

[B134] Ismail Z. Z. Tezel U. Pavlostathis S. G. (2010). Sorption of quaternary ammonium compounds to municipal sludge. Water Res. 44, 2303–13. 10.1016/j.watres.2009.12.029 20045549

[B135] Iyer A. P. Xue J. Honda M. Robinson M. Kumosani T. A. Abulnaja K.. (2017). Urinary levels of triclosan and triclocarban in several Asian countries, Greece and the USA: Association with oxidative stress. Environ. Res. 160, 91–6. 10.1016/j.envres.2017.09.021 28964967

[B136] Jang H. J. Chang M. W. Toghrol F. Bentley W. E. (2008). Microarray analysis of toxicogenomic effects of triclosan on *Staphylococcus aureus*. Appl. Microbiol. Biotechnol. 78, 695–707. 10.1007/s00253-008-1349-x 18210102

[B137] Jarusutthirak C. Sangsawang K. Mattaraj S. Jiraratananon R. (2012). Treatment of formaldehyde-containing wastewater using membrane bioreactor. J. Environ. Eng. 138, 265–271. 10.1061/(ASCE)EE.1943-7870.000043011848340

[B138] Jensen J. E. (1975). The effect of chlorhexidine on the anaerobic fermentation of Saccharomyces Cerevisiae. Biochem. Pharmacol. 24, 2163–2166. 10.1016/0006-2952(75)90047-7 1108884

[B139] Jolibois B. Guerbet M. Vassal S. (2002). Glutaraldehyde in hospital wastewater. Arch. Environ. Contam. Toxicol. 42, 137–144. 10.1007/s00244-001-0011-8 11815804

[B140] Jordan S. L. P. Russo M. R. Blessing R. L. Theis A. B. (1996). Inactivation of glutaraldehyde by reaction with sodium bisulfite. J. Toxicol. Environ. Heal - Part A. 47, 299–309. 10.1080/009841096161807 8604152

[B141] Joswick H. l. Corner T. R. Silvernale J. N. Gerhardt P. (1971). Antimicrobial actions of hexachlorophene release of cytoplasmic materials. J Bacteriol. 108, 492–500. 10.1128/jb.108.1.492-500.1971 4330741 PMC247090

[B142] Judis J. (1965). Mechanism of action of phenolic disinfectants IV. Effects on induction of and accessibility of substrate to β-galactosidase in *Escherichia coli*. J. Pharm. Sci. 54, 417–20. 10.1002/jps.2600540315 14301572

[B143] Judis J. (1966). Mechanism of action of phenolic disinfectants VII. J. Pharm. Sci. 55, 803–807. 10.1002/jps.2600550810 4166678

[B144] Juncker J. (2015). Commission Implementing Regulation (EU) 2015/1759 of 28 September 2015 approving glutaraldehyde as an existing active substance for use in biocidal products for product- types 2, 3. 4, 6, 11 and 12. Off. J. Eur. Union. L 257, 19–26.

[B145] Jury K. L. Vancov T. Stuetz R. M. Khan S. J. (2010). Antibiotic resistance dissemination and sewage treatment plants. Appl. Microbiol. 1, 509–519. Available online at: https://www.researchgate.net/publication/233427415_Antibiotic_resistance_dissemination_and_sewage_treatment_plants (accessed November 28, 2022).

[B146] Kaegi R. Voegelin A. Ort C. Sinnet B. Thalmann B. Krismer J.. (2013). Fate and transformation of silver nanoparticles in urban wastewater systems. Water Res. 47, 3866–77. 10.1016/j.watres.2012.11.060 23571111

[B147] Kampf G. (2018a). Biocidal agents used for disinfection can enhance antibiotic resistance in gram-negative species. Antibiotics. 7, 110. 10.3390/antibiotics7040110 30558235 PMC6316403

[B148] Kampf G. (2018b). Antiseptic Stewardship. London, UK: Springer Nature. 10.1007/978-3-319-98785-9

[B149] Kang H. I. Shin H. S. (2016). Determination of glutaraldehyde in water samples by headspace solid-phase microextraction and gas chromatography-mass spectrometry after derivatization with 2,2,2-trifluoroethylhydrazine. J. Chromatogr A. 1448, 115–20. 10.1016/j.chroma.2016.04.049 27130584

[B150] Karatzas K. A. G. Randall L. P. Webber M. Piddock L. J. V. Humphrey T. J. Woodward M. J.. (2008). Phenotypic and proteomic characterization of multiply antibiotic-resistant variants of *Salmonella enterica* serovar typhimurium selected following exposure to disinfectants. Appl. Environ. Microbiol. 74, 1508–1516. 10.1128/AEM.01931-07 18083849 PMC2258635

[B151] Karatzas K. A. G. Webber M. A. Jorgensen F. Woodward M. J. Piddock L. J. V. Humphrey T. J.. (2007). Prolonged treatment of *Salmonella enterica* serovar Typhimurium with commercial disinfectants selects for multiple antibiotic resistance, increased efflux and reduced invasiveness. J. Antimicrob. Chemother. 60, 947–955. 10.1093/jac/dkm314 17855722

[B152] Karkman A. Do T. T. Walsh F. Virta M. P. J. (2018). Antibiotic-resistance genes in waste water. Trends Microbiol. 26, 220–8. 10.1016/j.tim.2017.09.005 29033338

[B153] Kasianowicz J. Benz R. McLaughlin S. (1984). The kinetic mechanism by which CCCP (carbonyl cyanide m-Chlorophenylhydrazone) transports protons across membranes. J. Membr. Biol. 82, 179–190. 10.1007/BF01868942 6096547

[B154] Kasprzyk-Hordern B. Dinsdale R. M. Guwy A. J. (2008). The occurrence of pharmaceuticals, personal care products, endocrine disruptors and illicit drugs in surface water in South Wales, UK. Water Res. 42, 3498–3518. 10.1016/j.watres.2008.04.026 18514758

[B155] Kasprzyk-Hordern B. Dinsdale R. M. Guwy A. J. (2009). The removal of pharmaceuticals, personal care products, endocrine disruptors and illicit drugs during wastewater treatment and its impact on the quality of receiving waters. Water Res. 43, 363–80. 10.1016/j.watres.2008.10.047 19022470

[B156] Kathuria D. Bankar A. A. Bharatam P. V. (2018). “What's in a structure?” The story of biguanides. J. Mol. Struct. 1152, 61–78. 10.1016/j.molstruc.2017.08.100

[B157] Kimbrough R. D. (1973). Review of the toxicity of hexachlorophene, including its neurotoxicity. J. Clin. Pharmacol. New Drugs. 13, 439–444. 10.1002/j.1552-4604.1973.tb00196.x 4206035

[B158] Klein M. Deforest A. (1983). Principles of viral inactivation, in Disinfection, Sterilization and Preservation. Philadelphia, PA: Lippincott Williams & Wilkins. p. 422–434.

[B159] Korukluoglu M. Sahan Y. Yigit A. (2006). The fungicidal efficacy of various commercial disinfectants used in the food industry. Ann. Microbiol. 56, 325–330. 10.1007/BF03175025

[B160] Kratky M. Vinsova J. (2011). Salicylanilide ester prodrugs as potential antimicrobial agents - a review. Curr. Pharm. Des. 17, 3494–3505. 10.2174/138161211798194521 22074422

[B161] Kreuzinger N. Fuerhacker M. Scharf S. Uhl M. Gans O. Grillitsch B.. (2007). Methodological approach towards the environmental significance of uncharacterized substances - quaternary ammonium compounds as an example. Desalination. 215, 209–222. 10.1016/j.desal.2006.10.036

[B162] Kümmerer K. (2001). Drugs in the environment: Emission of drugs, diagnostic aids and disinfectants into wastewater by hospitals in relation to other sources - a review. Chemosphere. 45, 957–969. 10.1016/S0045-6535(01)00144-8 11695619

[B163] Kümmerle N. Feucht H. H. Kaulfers P. M. (1996). Plasmid-mediated formaldehyde resistance in *Escherichia coli*: characterization of resistance gene. Antimicrob. Agents Chemother. 40, 2276–2279. 10.1128/AAC.40.10.2276 8891129 PMC163518

[B164] Kuruvilla J. R. Kamath M. P. (1998). Antimicrobial activity of 2.5% sodium hypochlorite and 0.2% chlorhexidine gluconate separately and combined, as endodontic irrigants. J. Endod. 24, 472–476. 10.1016/S0099-2399(98)80049-6 9693573

[B165] Lachapelle J. M. Castel O. Casado A. F. Leroy B. Micali G. Tennstedt D.. (2013). Antiseptics in the era of bacterial resistance: a focus on povidone iodine. Clin. Pract. 10, 579–592. 10.2217/cpr.13.50

[B166] Lambert P. A. (2002). Cellular impermeability and uptake of biocides and antibiotics in Gram-positive bacteria and mycobacteria. J. Appl. Microbiol. Symp. Suppl. 92, 46–54. 10.1046/j.1365-2672.92.5s1.7.x 12000612

[B167] Langenhoff A. (2011). Shale Gas and Groundwater Quality: A Literature Review on Fate and Effects of Added Chemicals. Delft: Deltares. p. 22.

[B168] Larras F. Billoir E. Scholz S. Tarkka M. Wubet T. Delignette-Muller M. L.. (2020). A multi-omics concentration-response framework uncovers novel understanding of triclosan effects in the chlorophyte Scenedesmus vacuolatus. J. Hazard Mater. 397, 122727. 10.1016/j.jhazmat.2020.122727 32361673

[B169] Le D. Krasnopeeva E. Sinjab F. Pilizota T. Kim M. (2021). Active efflux leads to heterogeneous dissipation of proton motive force by protonophores in bacteria. MBio. 12, e0067621. 10.1128/mBio.00676-21 34253054 PMC8406135

[B170] Lee J. W. Cha D. K. Oh Y. K. Ko K. B. Song J. S. (2009). Zero-valent iron pretreatment for detoxifying iodine in liquid crystal display (LCD) manufacturing wastewater. J. Hazard. Mater. 164, 67–72. 10.1016/j.jhazmat.2008.07.147 18799266

[B171] Leggett M. J. Mcdonnell G. Denyer S. P. Setlow P. Maillard J. Y. (2012). Bacterial spore structures and their protective role in biocide resistance. J. Appl. Microbiol. 113, 485–498. 10.1111/j.1365-2672.2012.05336.x 22574673

[B172] Legin G. Y. (1996). 2-Bromo-2-nitro-1,3-propanediol (bronopol) and its derivatives: Synthesis, properties, and application (a review). Pharm. Chem. J. 30, 273–284. 10.1007/BF02218777

[B173] Lehutso R. F. Daso A. P. Okonkwo J. O. (2017). Occurrence and environmental levels of triclosan and triclocarban in selected wastewater treatment plants in Gauteng Province, South Africa. Emerg. Contam. 3, 107–14. 10.1016/j.emcon.2017.07.001

[B174] Leive L. (1965). Release of lipopolysaccharide by EDTA treatment of *E.Coli*. Biochem. Biophys. Res. Commun. 21, 290–296. 10.1016/0006-291X(65)90191-9 4159978

[B175] Lepelletier D. Maillard J. Y. Pozetto B. Simon A. (2020). Povidone iodine: properties, mechanisms of action, and role in infection control and *Staphylococcus aureus* decolonization. Oral Oncol. 105, 581–593. 10.1128/AAC.00682-20 32571829 PMC7449185

[B176] Leriche V. Briandet R. Carpentier B. (2003). Ecology of mixed biofilms subjected daily to a chlorinated alkaline solution: Spatial distribution of bacterial species suggests a protective effect of one species to another. Environ. Microbiol. 5, 64–71. 10.1046/j.1462-2920.2003.00394.x 12542714

[B177] Leung H. W. (2001). Ecotoxicology of glutaraldehyde: review of environmental fate and effects studies. Ecotoxicol. Environ. Saf. 49, 26–39. 10.1006/eesa.2000.2031 11386713

[B178] Levin B. C. Freese E. (1977). Comparison of the effects of two lipophilic acids, hexachlorophene and decanoate, on *Bacillus subtilis*. Antimicrob. Agents Chemother. 12, 357–367. 10.1128/AAC.12.3.357 410363 PMC429919

[B179] Li L. Hartmann G. Döblinger M. Schuster M. (2013). Quantification of nanoscale silver particles removal and release from municipal wastewater treatment plants in Germany. Environ. Sci. Technol. 47, 7317–7323. 10.1021/es3041658 23750458

[B180] Li L. Ye L. Kromann S. Meng H. (2017). Occurrence of extended-spectrum β-lactamases, plasmid-mediated quinolone resistance, and disinfectant resistance genes in *Escherichia coli* isolated from ready-to-eat meat products. Foodborne Pathog. Dis. 14, 109–115. 10.1089/fpd.2016.2191 27870554

[B181] Liberti L. Notarnicola M. (1999). Advanced treatment and disinfection for municipal wastewater reuse in agriculture. Water Sci. Technol. 40, 235–45. 10.1016/S0273-1223(99)00505-327450254

[B182] Lim S. J. Seo C. K. Kim T. H. Myung S. W. (2013). Occurrence and ecological hazard assessment of selected veterinary medicines in livestock wastewater treatment plants. J. Environ. Sci. Heal. - Part B Pestic Food Contam. Agric. Wastes. 48, 658–670. 10.1080/03601234.2013.778604 23638893

[B183] Lin S. H. Lin C. M. Leu H. G. (1999). Operating characteristics and kinetic studies of surfactant wastewater treatment by fenton oxidation. Water Res. 33, 1735–1741. 10.1016/S0043-1354(98)00403-5

[B184] Llompart M. Lourido M. Landín P. García-Jares C. Cela R. (2002). Optimization of a derivatization—solid-phase microextraction method for the analysis of thirty phenolic pollutants in water samples. J. Chromatogr. A. 963, 137–148. 10.1016/S0021-9673(02)00646-5 12187964

[B185] Lloyd W. J. Broadhurst A. V. Hall M. J. Andrews K. J. M. Barber W. E. Wong-Kai-In P.. (1988). Cyclohexane triones, novel membrane-active antibacterial agents. Antimicrob. Agents Chemother. 32, 814–818. 10.1128/AAC.32.6.814 3137860 PMC172288

[B186] Lopachin R. M. Gavin T. (2014). Molecular mechanisms of aldehyde toxicity: a chemical perspective. Chem. Res. Toxicol. 27, 1081–1091. 10.1021/tx5001046 24911545 PMC4106693

[B187] López-García E. Pérez-López C. Postigo C. Andreu V. Bijlsma L. González-Mariño I.. (2020). Assessing alcohol consumption through wastewater-based epidemiology: spain as a case study. Drug Alcohol Depend. 215, 108241. 10.1016/j.drugalcdep.2020.108241 32892109

[B188] Loshon C. A. Genest P. C. Setlow B. Setlow P. (1999). Formaldehyde kills spores of *Bacillus subtilis* by DNA damage and small, acid-soluble spore proteins of the α/β-type protect spores against this DNA damage. J. Appl. Microbiol. 87, 8–14. 10.1046/j.1365-2672.1999.00783.x 10432583

[B189] Lowbury E. J. L. Lilly H. A. (1960). Disinfection of the hands of surgeons and nurses. Br. Med. J. 1, 1445–1450. 10.1136/bmj.1.5184.1445 14418523 PMC1967299

[B190] Lozano N. Rice C. P. Ramirez M. Torrents A. (2013). Fate of Triclocarban, Triclosan and Methyltriclosan during wastewater and biosolids treatment processes. Water Res. 47, 4519–27. 10.1016/j.watres.2013.05.015 23764601

[B191] Lu G. Wu D. Fu R. (2007). Studies on the synthesis and antibacterial activities of polymeric quaternary ammonium salts from dimethylaminoethyl methacrylate. React. Funct. Polym. 67, 355–366. 10.1016/j.reactfunctpolym.2007.01.008

[B192] Luo Q. Wang J. Wang J. H. Shen Y. Yan P. Chen Y. P.. (2019). Fate and occurrence of pharmaceutically active organic compounds during typical pharmaceutical wastewater treatment. J Chem. 2019, 12. 10.1155/2019/2674852

[B193] Machado I. Coquet L. Jouenne T. Pereira M. O. (2013). Proteomic approach to *Pseudomonas aeruginosa* adaptive resistance to benzalkonium chloride. J. Proteomics. 89, 273–9. 10.1016/j.jprot.2013.04.030 23651563

[B194] Macomber L. Rensing C. Imlay J. A. (2007). Intracellular copper does not catalyze the formation of oxidative DNA damage in *Escherichia coli*. J. Bacteriol. 189, 1616–1626. 10.1128/JB.01357-06 17189367 PMC1855699

[B195] Maillard J. Y. (2002). Bacterial target sites for biocide action. J. Appl. Microbiol. Symp. Suppl. 92, 16–27. 10.1046/j.1365-2672.92.5s1.3.x 12000609

[B196] Maksym K. Gmur T. M. K. (2020). Povidone-iodine in wound healing and prevention of wound infections. Eur. J. Biol. Res. 10, 232–239. 10.5281/zenodo.3958220

[B197] Manzoor S. E. Lambert P. A. Griffiths P. A. Gill M. J. Fraise A. P. (1999). Reduced glutaraldehyde susceptibility in Mycobacterium chelonae associated with altered cell wall polysaccharides. J. Antimicrob. Chemother. 4, 759–765. 10.1093/jac/43.6.759 10404314

[B198] Margot J. Rossi L. Barry D. A. Holliger C. (2015). A review of the fate of micropollutants in wastewater treatment plants. WIREs Water. 2, 457–487. 10.1002/wat2.1090

[B199] Marzulli F. N. Bruch M. (1981). Antimicrobial soaps: benefits versus risks. Ski Microbiol. 1st edition, 125–34. 10.1007/978-1-4612-5868-1_1617683018

[B200] Mastrine J. A. Bonzongo J. C. J. Lyons W. B. (1999). Mercury concentrations in surface waters from fluvial systems draining historical precious metals mining areas in southeastern U.S.A. Appl. Geochemistry. 14, 147–158. 10.1016/S0883-2927(98)00043-2

[B201] Masuda N. Gotoh N. Ohya S. Nishino T. (1996). Quantitative correlation between susceptibility and OprJ production in NfxB mutants of *Pseudomonas aeruginosa*. Antimicrob. Agents Chemother. 40, 909–913. 10.1128/AAC.40.4.909 8849250 PMC163229

[B202] Masuda N. Sakagawa E. Ohya S. (1995). Outer membrane proteins responsible for multiple drug resistance in *Pseudomonas aeruginosa*. Antimicrob. Agents Chemother. 39, 645–649. 10.1128/AAC.39.3.645 7793866 PMC162598

[B203] Matsushima H. Sakurai N. A. (1984). selected ion monitoring assay for chlorhexidine in medical waste water. Biol. Mass Spectrom. 11, 203–206. 10.1002/bms.1200110502

[B204] Mc Carlie S. Boucher C. E. Bragg R. R. (2020). Molecular basis of bacterial disinfectant resistance. Drug Resist. Updat. 48, 100672. 10.1016/j.drup.2019.100672 31830738

[B205] Mc Cay P. H. Ocampo-Sosa A. A. Fleming G. T. A. (2010). Effect of subinhibitory concentrations of benzalkonium chloride on the competitiveness of *Pseudomonas aeruginosa* grown in continuous culture. Microbiology. 156, 30–38. 10.1099/mic.0.029751-0 19815578

[B206] McBain A. J. Ledder R. G. Moore L. E. Catrenich C. E. Gilbert P. (2004). Effects of quaternary-ammonium-based formulations on bacterial community dynamics and antimicrobial susceptibility. Appl. Environ. Microbiol. 70, 3449–3456. 10.1128/AEM.70.6.3449-3456.2004 15184143 PMC427781

[B207] McDonnell G. (2009). Sterilization and disinfection. in Encyclopedia of Microbiology, 3 ed. M. Schaechter. (San Diego, CA), 529–48.

[B208] Mcdonnell G. Russell A. D. (1999). Antiseptics and disinfectants: Activity, action, and resistance. Clin. Microbiol. Rev. 12, 147–179. 10.1128/CMR.12.1.147 9880479 PMC88911

[B209] McLaughlin S. G. A. Dilger J. P. (1980). Transport of protons across membranes by weak acids. Physiol. Rev. 60, 825–863. 10.1152/physrev.1980.60.3.825 6248908

[B210] McMurry L. M. Oethinger M. Levy S. B. (1998). Overexpression of marA, soxS, or acrAB produces resistance to triclosan in laboratory and clinical strains of *Escherichia coli*. FEMS Microbiol. Lett. 166, 305–309. 10.1111/j.1574-6968.1998.tb13905.x 9770288

[B211] Merchel Piovesan Pereira B. Wang X. Tagkopoulos I. (2021). Biocide-induced emergence of antibiotic resistance in *Escherichia coli*. Front. Microbiol. 12, 1–12. 10.3389/fmicb.2021.640923 33717036 PMC7952520

[B212] Merkel T. H. Gro,ß H. J. Werner W. Dahlke T. Reicherter S. Beuchle G.. (2002). Copper corrosion by-product release in long-term stagnation experiments. Water Res. 36, 1547–1555. 10.1016/S0043-1354(01)00366-9 11996343

[B213] Migneault K. C. W. (2004). Glutaraldehyde: behavior in aqueous solution, reaction with proteins, and application to enzyme crosslinking. J. Mater Chem. A. 4, 18687–18705. 10.2144/04375RV01 15560135

[B214] Mijnendonckx K. Leys N. Mahillon J. Silver S. Van Houdt R. (2013). Antimicrobial silver: uses, toxicity and potential for resistance. Biometals. 26, 609–621. 10.1007/s10534-013-9645-z 23771576

[B215] Mima T. Joshi S. Gomez-Escalada M. Schweizer H. P. (2007). Identification and characterization of TriABC-OpmH, a triclosan efflux pump of *Pseudomonas aeruginosa* requiring two membrane fusion proteins. J. Bacteriol. 189, 7600–7609. 10.1128/JB.00850-07 17720796 PMC2168734

[B216] Mitchell P. (2011). Chemiosmotic coupling in oxidative and photosynthetic phosphorylation. Biochim. Biophys. Acta - Bioenerg. 1807, 1507–38. 10.1016/j.bbabio.2011.09.018 22082452

[B217] Modak S. M. Fox C. L. (1973). Binding of silver sulfadiazine to the cellular components of *Pseudomonas aeruginosa*. Biochem. Pharmacol. 22, 2391–2404. 10.1016/0006-2952(73)90341-9 4200887

[B218] Modi S. R. Lee H. H. Spina C. S. Collins J. J. (2013). Antibiotic treatment expands the resistance reservoir and ecological network of the phage metagenome. Nature. 499, 219–222. 10.1038/nature12212 23748443 PMC3710538

[B219] Moen B. Rudi K. Bore E. Langsrud S. (2012). Subminimal inhibitory concentrations of the disinfectant benzalkonium chloride select for a tolerant subpopulation of *Escherichia coli* with inheritable characteristics. Int. J. Mol. Sci. 13, 4101–4123. 10.3390/ijms13044101 22605968 PMC3344204

[B220] Moran J. E. Oktay S. D. Santschi P. H. (2002). Sources of iodine and iodine 129 in rivers. Water Resour. Res. 38, 24–1.-24–10. 10.1029/2001WR000622

[B221] Morante J. Quispe A. M. Ymaña B. Moya-Salazar J. Luque N. Soza G.. (2021). Tolerance to disinfectants (chlorhexidine and isopropanol) and its association with antibiotic resistance in clinically-related Klebsiella pneumoniae isolates. Pathog. Glob Health. 115, 53–60. Available from: 10.1080/20477724.2020.1845479 33455564 PMC7850358

[B222] Møretr,ø T. Schirmer B. C. T. Heir E. Fagerlund A. Hjemli P. Langsrud S.. (2017). Tolerance to quaternary ammonium compound disinfectants may enhance growth of Listeria monocytogenes in the food industry. Int. J. Food Microbiol. 241, 215–24. 10.1016/j.ijfoodmicro.2016.10.025 27810443

[B223] Morita Y. Murata T. Mima T. Shiota S. Kuroda T. Mizushima T.. (2003). Induction of mexCD-oprJ operon for a multidrug efflux pump by disinfectants in wild-type *Pseudomonas aeruginosa* PAO1. J. Antimicrob. Chemother. 51, 991–994. 10.1093/jac/dkg173 12654738

[B224] Morrison L. Zembower T. R. (2020). Antimicrobial resistance - global report on surveillance. Gastrointest. Endosc. Clin. N. Am. 30, 619–635. 10.1016/j.giec.2020.06.004 32891221

[B225] Morton H. E. (1950). the Relationship of concentration and germicidal efficiency of ethyl alcohol. Ann. N. Y. Acad. Sci. 53, 191–196. 10.1111/j.1749-6632.1950.tb31944.x 15433175

[B226] Murphy E. C. Friedman A. J. (2019). Hydrogen peroxide and cutaneous biology: Translational applications, benefits, and risks. J. Am. Acad. Dermatol. 81, 1379–86. 10.1016/j.jaad.2019.05.030 31103570

[B227] Musee N. (2018). Environmental risk assessment of triclosan and triclocarban from personal care products in South Africa. Environ. Pollut. 242, 827–38. 10.1016/j.envpol.2018.06.106 30036836

[B228] National Center for Biotechnology Information. (2004a). PubChem Compound Summary for CID 8758, Nitrilotriacetic acid. Available online at: https://pubchem.ncbi.nlm.nih.gov/compound/Nitrilotriacetic-acid (accessed November 9, 2022).

[B229] National Center for Biotechnology Information. (2004b). PubChem Compound Summary for CID 6049, Edetic acid. Available online at: https://pubchem.ncbi.nlm.nih.gov/compound/Edetic-acid (accessed November 9, 2022).

[B230] Neal T. (1973). Age differences in Susceptibility of Swiss White Mice to Hexachlorophene Toxicity. Available online at: https://shareok.org/bitstream/handle/11244/23898/Thesis-1973-N343a.pdf?sequence=1 (accessed Nov 28, 2022).

[B231] Nhung N. T. Thuy C. T. Trung N. V. Campbell J. Baker S. Thwaites G.. (2015). Induction of antimicrobial resistance in *Escherichia coli* and non-typhoidal *Salmonella strains* after adaptation to disinfectant commonly used on farms in Vietnam. Antibiotics. 4, 480–494. 10.3390/antibiotics4040480 27025637 PMC4790309

[B232] Nicolae Dopcea G. Dopcea I. Nanu A. E. (2020). Digută CF, Matei F. Resistance and cross-resistance in Staphylococcus spp. strains following prolonged exposure to different antiseptics. J Glob Antimicrob Resist. 21, 399–404. 10.1016/j.jgar.2019.10.021 31698107

[B233] Nomura K. Ogawa M. Miyamoto H. Muratani T. Taniguchi H. (2004). Antibiotic susceptibility of glutaraldehyde-tolerant Mycobacterium chelonae from bronchoscope washing machines. Am. J. Infect. Control. 32, 185–188. 10.1016/j.ajic.2003.07.007 15175610

[B234] Norman G. Christie J. Liu Z. Westby M. J. Jefferies J. M. Hudson T.. (2017). Antiseptics for burns. Cochrane Database Syst. Rev. 2017, 12. 10.1002/14651858.CD011821.pub2 28700086 PMC6483239

[B235] Noyce J. O. Michels H. Keevil C. W. (2007). Inactivation of influenza A virus on copper versus steel surfaces. Am Soc Microbiol. 73, 2748–50. 10.1128/AEM.01139-06 17259354 PMC1855605

[B236] Obłak E. Futoma-Kołoch B. Wieczyńska A. (2021). Biological activity of quaternary ammonium salts and resistance of microorganisms to these compounds. World J. Microbiol. Biotechnol. 37, 1–11. Available from: 10.1007/s11274-020-02978-0 33428020

[B237] Ortegón L. Puentes-Herrera M. Corrales I. F. Cortés J. A. (2017). Colonization and infection in the newborn infant: does chlorhexidine play a role in infection prevention. Arch. Argent. Pediatr. 115, 65–70. 10.5546/aap.2017.eng.65 28097843

[B238] Orth R. (1998). The importance of disinfection for the hygiene in the dairy and beverage production. Int. Biodeterior. Biodegrad. 41, 201–208. 10.1016/S0964-8305(98)00036-5

[B239] Östman M. Fick J. Tysklind M. (2018). Detailed mass flows and removal efficiencies for biocides and antibiotics in Swedish sewage treatment plants. Sci. Total Environ. (2018) 640–41, 327–36. 10.1016/j.scitotenv.2018.05.304 29860006

[B240] Östman M. Lindberg R. H. Fick J. Björn E. Tysklind M. (2017). Screening of biocides, metals and antibiotics in Swedish sewage sludge and wastewater. Water Res. 115, 318–328. 10.1016/j.watres.2017.03.011 28288311

[B241] Pál C. Papp B. Lercher M. J. (2005). Adaptive evolution of bacterial metabolic networks by horizontal gene transfer. Nat. Genet. 37, 1372–1375. 10.1038/ng1686 16311593

[B242] Pang X. Wong C. Chung H. J. Yuk H. G. (2019). Biofilm formation of Listeria monocytogenes and its resistance to quaternary ammonium compounds in a simulated salmon processing environment. Food Control. 98, 200–8. 10.1016/j.foodcont.2018.11.029

[B243] Pati S. G. Arnold W. A. (2020). Comprehensive screening of quaternary ammonium surfactants and ionic liquids in wastewater effluents and lake sediments. Environ. Sci. Process. Impacts. 22, 430–441. 10.1039/C9EM00554D 32003378

[B244] Peña M. M. O. Lee J. Thiele D. J. A. (1999). Delicate balance: homeostatic control of copper uptake and distribution. J. Nutr. 129, 1251–1260. 10.1093/jn/129.7.1251 10395584

[B245] Percival S. L. Kite P. Eastwood K. Murga R. Carr J. Arduino M. J.. (2005). Tetrasodium EDTA as a novel central venous catheter lock solution against biofilm. Infect. Control Hosp. Epidemiol. 26, 515–519. 10.1086/502577 16018425

[B246] Pereira B. M. P. Tagkopoulos I. (2019). Benzalkonium chlorides: uses, regulatory status, and microbial resistance. Appl. Environ. Microbiol. 85, 1–13. 10.1128/AEM.00377-19 31028024 PMC6581159

[B247] Pérez-Alvarez I. Islas-Flores H. Gómez-Oliván L. M. Barcel,ó D. López De Alda M. Pérez Solsona S.. (2018). Determination of metals and pharmaceutical compounds released in hospital wastewater from Toluca, Mexico, and evaluation of their toxic impact. Environ. Pollut. 240, 330–341. 10.1016/j.envpol.2018.04.116 29751329

[B248] Piddock L. J. V. (2006). Multidrug-resistance efflux pumps-not just for resistance. Nat. Rev. Microbiol. 4, 629–636. 10.1038/nrmicro1464 16845433

[B249] Polesel F. Farkas J. Kjos M. Almeida Carvalho P. Flores-Alsina X. Gernaey K. V.. (2018). Occurrence, characterisation and fate of (nano)particulate Ti and Ag in two Norwegian wastewater treatment plants. Water Res. 141, 19–31. 10.1016/j.watres.2018.04.065 29753974

[B250] Poole K. (2007). Efflux pumps as antimicrobial resistance mechanisms. Ann. Med. 39, 162–176. 10.1080/07853890701195262 17457715

[B251] Puangseree J. Jeamsripong S. Prathan R. Pungpian C. Chuanchuen R. (2021). Resistance to widely-used disinfectants and heavy metals and cross resistance to antibiotics in *Escherichia coli* isolated from pigs, pork and pig carcass. Food Control. 124, 107892. 10.1016/j.foodcont.2021.107892

[B252] Rajamohan G. Srinivasan V. B. Gebreyes W. A. (2009). Novel role of Acinetobacter baumannii RND efflux transporters in mediating decreased susceptibility to biocides. J. Antimicrob. Chemother. 65, 228–232. 10.1093/jac/dkp427 20008046

[B253] Randall L. P. Cooles S. W. Coldham N. G. Penuela E. G. Mott A. C. Woodward M. J.. (2007). Commonly used farm disinfectants can select for mutant *Salmonella enterica* serovar Typhimurium with decreased susceptibility to biocides and antibiotics without compromising virulence. J. Antimicrob. Chemother. 60, 1273–1280. 10.1093/jac/dkm359 17897935

[B254] Report A. (2012). Alkyl (C 12-16) dimethylbenzyl ammonium chloride. p. 8.

[B255] Rosenkranz H. Rosenkranz S. (1972). Silver sulfadiazine: interaction with isolated deoxyribonucleic acid. Am. Soc. Microbiol. 2, 373–383. 10.1128/AAC.2.5.373 4677596 PMC444323

[B256] Rosenthal R. A. Dassanayake N. L. Schlitzer R. L. Schlech B. A. Meadows D. L. Stone R. P.. (2006). Biocide uptake in contact lenses and loss of fungicidal activity during storage of contact lenses. Eye Contact Lens. 32, 262–266. 10.1097/ICL.0b013e31802b413f 17099385

[B257] Russell A. D. (2004). Bacterial adaptation and resistance to antiseptics, disinfectants and preservatives is not a new phenomenon. J. Hosp. Infect. 57, 97–104. 10.1016/j.jhin.2004.01.004 15183238

[B258] Russell H.ugo, Ayliffe's. (2013). Principles and Practice of Disinfection, Preservation and Sterilization. 5th ed. Fraise A. P Maillard J. Y. Sattar S. A. (eds). Wiley-Blackwell. p. 616.

[B259] Rutala W. A. Weber D. J. (2001). New disinfection and sterilization methods. Emerging Infect. Dis. 7, 348–353. 10.3201/eid0702.010241 11294738 PMC2631727

[B260] Salaudeen T. Okoh O. Okoh A. (2019). Performance assessment of wastewater treatment plants with special reference to phenol removal. Int. J. Environ. Sci. Technol. 16, 401–12. 10.1007/s13762-018-1684-0

[B261] Santana C. M. Ferrera Z. S. Padrón M. E. T. Rodríguez J. J. S. (2009). Methodologies for the extraction of phenolic compounds from environmental samples: new approaches. Molecules. 14, 298–320. 10.3390/molecules14010298 19136918 PMC6253767

[B262] Sasatsu M. Shirai Y. Hase M. Noguchi N. Kono M. Behr H.. (1995). The origin of the antiseptic-resistance gene ebr in *Staphylococcus aureus*. Microbios. 84, 161–169. 8820242

[B263] Schlüter A. Szczepanowski R. Pühler A. Top E. M. (2007). Genomics of IncP-1 antibiotic resistance plasmids isolated from wastewater treatment plants provides evidence for a widely accessible drug resistance gene pool. FEMS Microbiol. Rev. 31, 449–477. 10.1111/j.1574-6976.2007.00074.x 17553065

[B264] Schreurs W. J. Rosenberg H. (1982). Effect of Silver Ions on transport and retention of phosphate byEcoli. J. Bacteriol. 152, 7–13. 10.1128/jb.152.1.7-13.1982 6749823 PMC221367

[B265] Shafer M. M. Overdier J. T. Armstong D. E. (1998). Removal, partitioning, and fate of silver and other metals in wastewater treatment plants and effluent-receiving streams. Environ. Toxicol. Chem. 17, 630–641. 10.1002/etc.5620170416

[B266] Shen J. Y. Chang M. S. Yang S. H. Wu G. J. (2012a). Simultaneous and rapid determination of triclosan, triclocarban and their four related transformation products in water samples using SPME-HPLC-DAD. J. Liq. Chromatogr. Relat. Technol. 35, 2280–2293. 10.1080/10826076.2011.631258

[B267] Shen J. Y. Chang M. S. Yang S. H. Wu G. J. (2012b). Simultaneous determination of triclosan, triclocarban, and transformation products of triclocarban in aqueous samples using solid-phase micro-extraction-HPLC-MS/MS. J. Sep. Sci. 35, 2544–2552. 10.1002/jssc.201200181 22907835

[B268] Shepherd J. A. Waigh R. D. Gilbert P. (1988). Antibacterial action of 2-bromo-2-nitropropane-1, 3-Diol *(Bronopol)*. 32, 1693–1698. 10.1128/AAC.32.11.1693 3075439 PMC175953

[B269] Silver S. (2003). Bacterial silver resistance: molecular biology and uses and misuses of silver compounds. FEMS Microbiol. Rev. 27, 341–353. 10.1016/S0168-6445(03)00047-0 12829274

[B270] Silvernale J. N. Joswick H. L. Corner T. R. Gerhardt P. (1971). Antimicrobial actions of hexachlorophene: cytological manifestations. J. Bacteriol. 108, 482–491. 10.1128/jb.108.1.482-491.1971 4107813 PMC247089

[B271] Singer H. Müller S. Tixier C. Pillonel L. (2002). Triclosan: Occurrence and fate of a widely used biocide in the aquatic environment: field measurements in wastewater treatment plants, surface waters, and lake sediments. Environ. Sci. Technol. 36, 4998–5004. 10.1021/es025750i 12523412

[B272] Small D. A. Chang W. Toghrol F. Bentley W. E. (2007). Comparative global transcription analysis of sodium hypochlorite, peracetic acid, and hydrogen peroxide on *Pseudomonas aeruginosa*. Appl. Microbiol. Biotechnol. 76, 1093–1105. 10.1007/s00253-007-1072-z 17624526

[B273] Smith R. (2015). Directive 98/5/EC of the European parliament and of the council of 16 February 1998. Core EU Legis. 41, 197–202. 10.1007/978-1-137-54482-7_22

[B274] Snyder L. Champness W. (2007). Molecular genetics of bacteria. ASM Press; p. 758.

[B275] Spataro F. Ademollo N. Pescatore T. Rauseo J. Patrolecco L. (2019). Antibiotic residues and endocrine disrupting compounds in municipal wastewater treatment plants in Rome, Italy. Microchem J. 148, 634–42. 10.1016/j.microc.2019.05.053

[B276] Sreevidya V. S. Lenz K. A. Svoboda K. R. Ma H. (2018). Benzalkonium chloride, benzethonium chloride, and chloroxylenol - three replacement antimicrobials are more toxic than triclosan and triclocarban in two model organisms. Environ. Pollut. 235, 814–24. 10.1016/j.envpol.2017.12.108 29348075

[B277] Stawarz-Janeczek M. Kryczyk-Poprawa A. Muszyńska B. Opoka W. Pytko-Polończyk J. (2021). Disinfectants used in stomatology and SARS-CoV-2 infection. Eur. J. Dent. 15, 388–400. 10.1055/s-0041-1724154 33694135 PMC8184310

[B278] Stout J. E. Yu V. L. (2014). Experiences of the first 16 hospitals using copper – silver ionization for legionella control : implications for the evaluation of other disinfection modalities. Infect. Control Hosp. Epidemiol. 24, 563–568. 10.1086/502251 12940575

[B279] Sun Q. Li M. Ma C. Chen X. Xie X. Yu C. P.. (2016). Seasonal and spatial variations of PPCP occurrence, removal and mass loading in three wastewater treatment plants located in different urbanization areas in Xiamen, China. Environ. Pollut. 208, 371–81. 10.1016/j.envpol.2015.10.003 26552527

[B280] Svetlíková Z. Škovierová H. Niederweis M. Gaillard J. L. McDonnell G. Jackson M.. (2009). Role of porins in the susceptibility of Mycobacterium smegmatis and Mycobacterium chelonae to aldehyde-based disinfectants and drugs. Antimicrob. Agents Chemother. 53, 4015–4018. 10.1128/AAC.00590-09 19581465 PMC2737867

[B281] Talaiekhozani A. Salari M. Talaei M. R. Bagheri M. Eskandari Z. (2016). Formaldehyde removal from wastewater and air by using UV, ferrate(VI) and UV/ferrate(VI). J. Environ. Manage. 184, 204–9. 10.1016/j.jenvman.2016.09.084 27717675

[B282] Taweetanawanit P. Ratpukdi T. Siripattanakul-Ratpukdi S. (2018). Performance and kinetics of triclocarban removal by entrapped Pseudomonas fluorescens strain MC46. Bioresour. Technol. 274, 113–9. 10.1016/j.biortech.2018.11.085 30502601

[B283] Tennent J. M. Lyon B. R. Midgley M. Jones I. G. Purewal A. S. Skurray R. A.. (1989). Physical and biochemical characterization of the qacA gene encoding antiseptic and disinfectant resistance in *Staphylococcus aureus*. J. Gen. Microbiol. 135, 1–10. 10.1099/00221287-135-1-1 2778425

[B284] Tezel U. Pavlostathis S. G. (2009). Transformation of benzalkonium chloride under nitrate reducing conditions. Environ. Sci. Technol. 43, 1342–1348. 10.1021/es802177f 19350901

[B285] Thomas C. M. (2000). Paradigms of plasmid organization. Mol. Microbiol. 37, 485–491. 10.1046/j.1365-2958.2000.02006.x 10931342

[B286] Thomas K. V. Thain J. E. Waldock M. J. (1999). Identification of toxic substances in United Kingdom estuaries. Environ. Toxicol. Chem. 18, 401–411. 10.1002/etc.562018030615091993

[B287] Tilley F. W. (1945). The influence of changes in concentration and temperature upon the bactericidal activity of formaldehyde in aqueous solutions. J. Bacteriol. 50, 469–473. 10.1128/jb.50.4.469-473.1945 21008210

[B288] Tischer M. Pradel G. Ohlsen K. Holzgrabe U. (2012). Quaternary ammonium salts and their antimicrobial potential: targets or nonspecific interactions? ChemMedChem. 7, 22–31. 10.1002/cmdc.201100404 22113995

[B289] Tkachenko O. Shepard J. Aris V. M. Joy A. Bello A. Londono I.. (2007). A triclosan-ciprofloxacin cross-resistant mutant strain of *Staphylococcus aureus* displays an alteration in the expression of several cell membrane structural and functional genes. Res. Microbiol. 158, 651–658. 10.1016/j.resmic.2007.09.003 17997080

[B290] Tong C. Hu H. Chen G. Li Z. Li A. Zhang J.. (2021). Disinfectant resistance in bacteria: mechanisms, spread, and resolution strategies. Environ. Res. 195, 110897. 10.1016/j.envres.2021.110897 33617866

[B291] Traor,é O. Allaert F. A. Fournet-Fayard S. Verrière J. L. Laveran H. (2000). Comparison of in-vivo antibacterial activity of two skin disinfection procedures for insertion of peripheral catheters: povidone iodine versus chlorhexidine. J. Hosp. Infect. 44, 147–150. 10.1053/jhin.1999.0685 10662566

[B292] Treangen T. J. Rocha E. P. C. (2011). Horizontal transfer, not duplication, drives the expansion of protein families in prokaryotes. PLoS Genet. 7, e1001284. 10.1371/journal.pgen.1001284 21298028 PMC3029252

[B293] Tripathi K. D. (2019). Essentials of Medical Pharmacology. Paper Knowledge, in Toward a Media History of Documents. New Dehli: Jaypee Brothers Medical Pub. p. 960.

[B294] U.S. Department of Health and Human Services PHS (2021). Report on Carcinogens, Fifteenth Edition: Formaldehyde. Natl Toxicol Program, in Dep. Heal. Hum. Serv. (2000). Available online at: http://ntp.niehs.nih.gov/go/roc (accessed November 28, 2022).

[B295] Uluseker C. Kaster K. M. Thorsen K. Basiry D. Shobana S. Jain M.. (2021). A review on occurrence and spread of antibiotic resistance in wastewaters and in wastewater treatment plants: mechanisms and perspectives. Front. Microbiol. 12, 717809. 10.3389/fmicb.2021.717809 34707579 PMC8542863

[B296] Usman M. Farooq M. Hanna K. (2020). Environmental side effects of the injudicious use of antimicrobials in the era of COVID-19. Sci. Total Environ. 745, 141053. 10.1016/j.scitotenv.2020.141053 32702547 PMC7368658

[B297] Vaara M. (1992). Agents that increase the permeability of the outer membrane. Microbiol. Rev. 56, 395–411. 10.1128/mr.56.3.395-411.1992 1406489 PMC372877

[B298] Varela A. R. Manaia C. M. (2013). Human health implications of clinically relevant bacteria in wastewater habitats. Environ. Sci Pollut. Res. 20, 3550–3569. 10.1007/s11356-013-1594-0 23508533

[B299] Verovšek T. Heath D. Heath E. (2022). Occurrence, fate and determination of tobacco (nicotine) and alcohol (ethanol) residues in waste- and environmental waters. Trends Environ. Anal Chem. 34, e00158. 10.1016/j.teac.2022.e00164

[B300] Villegas L. G. C. Mashhadi N. Chen M. Mukherjee D. Taylor K. E. Biswas N. A.. (2016). Short review of techniques for phenol removal from wastewater. Curr. Pollut. Reports. 2, 157–67. 10.1007/s40726-016-0035-319833317

[B301] Vimalkumar K. Arun E. Krishna-Kumar S. Poopal R. K. Nikhil N. P. Subramanian A.. (2018). Occurrence of triclocarban and benzotriazole ultraviolet stabilizers in water, sediment, and fish from Indian rivers. Sci. Total Environ. 625, 1351–60. 10.1016/j.scitotenv.2018.01.042 29996432

[B302] Walia K. Argüello H. Lynch H. Grant J. Leonard F. C. Lawlor P. G.. (2017). The efficacy of different cleaning and disinfection procedures to reduce Salmonella and Enterobacteriaceae in the lairage environment of a pig abattoir. Int. J. Food Microbiol. 246, 64–71. 10.1016/j.ijfoodmicro.2017.02.002 28189901

[B303] Walsh S. E. Maillard J.-., Y. Russell A. D. Hann A. C. (2001). Possible mechanisms for the relative efficacies of ortho phthalaldehyde and glutaraldehyde agaisnt glutaraldehyde resistant mycobacterium chelonae. J. Appl. Microbiol. 91, 80–92. 10.1046/j.1365-2672.2001.01341.x 11442717

[B304] Walsh S. E. Maillard J.-., Y. Simons C. Russell A. D. (1999). Studies on the mechanisms of the antibacterial action of ortho-phthalaldehyde. J. Appl. Microbiol. 87, 702–10. 10.1046/j.1365-2672.1999.00913.x 10594711

[B305] Walsh S. E. Maillard J. Y. Russell A. D. Catrenich C. E. Charbonneau D. L. Bartolo R. G.. (2003). Activity and mechanisms of action of selected biocidal agents on Gram-positive and -negative bacteria. J. Appl. Microbiol. 94, 240–247. 10.1046/j.1365-2672.2003.01825.x 12534815

[B306] Wang Q. Kim D. Dionysiou D. D. Sorial G. A. Timberlake D. (2004). Sources and remediation for mercury contamination in aquatic systems - a literature review. Environ. Pollut. 131, 323–336. 10.1016/j.envpol.2004.01.010 15234099

[B307] Wang X. Wu P. Lv Y. Hou X. (2011). Ultrasensitive fluorescence detection of glutaraldehyde in water samples with bovine serum albumin-Au nanoclusters. Microchem. J. 99, 327–331. 10.1016/j.microc.2011.06.004

[B308] Wang Y. Teng Y. Wang D. Han K. Wang H. Kang L.. (2020). The fate of triclocarban in activated sludge and its influence on biological wastewater treatment system. J. Environ. Manage. 276, 111237. 10.1016/j.jenvman.2020.111237 32866751

[B309] Watras C. J. Morrison K. A. Host J. S. Bloom N. S. (1995). Concentration of mercury species in relationship to other site-specific factors in the surface waters of northern Wisconsin lakes. Limnol. Oceanogr. 40, 556–565. 10.4319/lo.1995.40.3.0556

[B310] Webber M. A. Coldham N. G. Woodward M. J. Piddock L. J. V. (2008). Proteomic analysis of triclosan resistance in *Salmonella enterica* serovar Typhimurium. J. Antimicrob. Chemother. 62, 92–97. 10.1093/jac/dkn138 18388111

[B311] Wiest L. Chonova T. Berg,é A. Baudot R. Bessueille-Barbier F. Ayouni-Derouiche L.. (2018). Two-year survey of specific hospital wastewater treatment and its impact on pharmaceutical discharges. Environ. Sci. Pollut. Res. 25, 9207–9218. 10.1007/s11356-017-9662-5 28718023

[B312] Wimmer A. Markus A. A. Schuster M. (2019). Silver nanoparticle levels in river water: real environmental measurements and modeling approaches - a comparative study. Environ. Sci. Technol. Lett. 6, 353–358. 10.1021/acs.estlett.9b00211

[B313] Xie Y. Chen L. Liu R. A. O. X. (2017). contamination status and genotoxicity of AOX-bearing pharmaceutical wastewater. J. Environ. Sci (China). 52, 170–7. 10.1016/j.jes.2016.04.014 28254035

[B314] Yan J. Liu J. Ren J. Wu Y. Li X. Sun T.. (2022). Design and multi-objective optimization of hybrid reactive-extractive distillation process for separating wastewater containing benzene and isopropanol. Sep. Purif Technol. 290, 120915. 10.1016/j.seppur.2022.120915

[B315] Yarley O. P. N. Kojo A. B. Zhou C. Yu X. Gideon A. Kwadwo H. H.. (2021). Reviews on mechanisms of in vitro antioxidant, antibacterial and anticancer activities of water-soluble plant polysaccharides. Int. J. Biol. Macromol. (2021) 183, 2262–71. 10.1016/j.ijbiomac.2021.05.181 34062158

[B316] Yasir M. Keith Turner A. Bastkowski S. Baker D. Page A. J. Telatin A.. (2020). TRADIS-XPress: a high-resolution whole-genome assay identifies novel mechanisms of triclosan action and resistance. Genome Res. 30, 239–249. 10.1101/gr.254391.119 32051187 PMC7050523

[B317] Ying G. G. Kookana R. S. (2007). Triclosan in wastewaters and biosolids from Australian wastewater treatment plants. Environ. Int. 33, 199–205. 10.1016/j.envint.2006.09.008 17055058

[B318] Yu J. T. Bouwer E. J. Coelhan M. (2006). Occurrence and biodegradability studies of selected pharmaceuticals and personal care products in sewage effluent. Agric Water Manag. 86, 72–80. 10.1016/j.agwat.2006.06.015

[B319] Yuan D. Tian L. Gu D. Shen X. Zhu L. Wu H.. (2017). Fast and efficient oxidation of formaldehyde in wastewater via the Solar Thermal Electrochemical Process tuned by thermo-electrochemistry. J. Clean. Prod. 156, 310–316. 10.1016/j.jclepro.2017.04.022

[B320] Yun H. Liang B. Kong D. Li X. Wang A. (2020). Fate, risk and removal of triclocarban: a critical review. J Hazard Mater. 387, 121944. 10.1016/j.jhazmat.2019.121944 31901847

[B321] Zhang C. Cui F. Zeng G. Jiang M. Yang Z. Yu Z.. (2015). Quaternary ammonium compounds (QACs): a review on occurrence, fate and toxicity in the environment. Sci. Total Environ. 518–519, 352–62. 10.1016/j.scitotenv.2015.03.007 25770948

[B322] Zhao J. L. Ying G. G. Wang L. Yang J. F. Yang X. B. Yang L. H.. (2009). Determination of phenolic endocrine disrupting chemicals and acidic pharmaceuticals in surface water of the Pearl Rivers in South China by gas chromatography-negative chemical ionization-mass spectrometry. Sci. Total Environ. 407, 962–74. 10.1016/j.scitotenv.2008.09.048 19004474

[B323] Zhao T. Chen Q. (2016). Halogenated phenols and polybiguanides as antimicrobial textile finishes. Antimicrobial Textiles. Elsevier Ltd. p. 141–153.

[B324] Zheng G. Filippelli G. M. Salamova A. (2020a). Increased indoor exposure to commonly used disinfectants during the COVID-19 pandemic. Environ. Sci. Technol. Lett. 7, 760–765. 10.1021/acs.estlett.0c00587 37566290 PMC7482546

[B325] Zheng G. Yu B. Wang Y. Ma C. Chen T. (2020b). Removal of triclosan during wastewater treatment process and sewage sludge composting—a case study in the middle reaches of the Yellow River. Environ Int. 134, 105300. 10.1016/j.envint.2019.105300 31726362

[B326] Zhong W. Wang D. Xu X. Luo Q. Wang B. Shan X.. (2010). Screening level ecological risk assessment for phenols in surface water of the Taihu Lake. Chemosphere. 80, 998–1005. 10.1016/j.chemosphere.2010.05.036 20557922

[B327] Zhou F. Li X. Zeng Z. (2005). Determination of phenolic compounds in wastewater samples using a novel fiber by solid-phase microextraction coupled to gas chromatography. Anal. Chim. Acta. 538, 63–70. 10.1016/j.aca.2005.02.009

[B328] Zhou T. Song Z. Zhang X. Gani R. Sundmacher K. (2019). Optimal solvent design for extractive distillation processes: a multiobjective optimization-based hierarchical framework. Ind. Eng. Chem. Res. 58, 5777–5786. 10.1021/acs.iecr.8b04245

[B329] Zinchenko A. A. Sergeyev V. G. Yamabe K. Murata S. Yoshikawa K. D. N. A. (2004). compaction by divalent cations: Structural specificity revealed by the potentiality of designed quaternary diammonium salts. Chembiochem. 5, 360–368. 10.1002/cbic.200300797 14997528

