# Supplementary material for: The effect of disinfectants and antiseptics on co- and cross-selection of resistance to antibiotics in aquatic environments and wastewater treatment plants
Source: Front Microbiol. 2022 Dec 13;13:1050558. doi: 10.3389/fmicb.2022.1050558 (PMC9793094; doi:10.3389/fmicb.2022.1050558)
Supplement: Supplementary file 1 [file Table_1.docx]

**Table 1:** Displayed are common representatives of different DA groups and their chemical structure.

| Compound | Structure | Function | Group | Ref. |
| --- | --- | --- | --- | --- |
| Ethanol, Isopropanol | 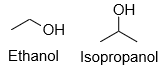 | Antisepsis, disinfection | Alcohol | (16) |
| Glutaraldehyde | 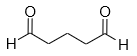 | Disinfection, sterilization | Aldehyde | (17-20) |
| Triclocarban | 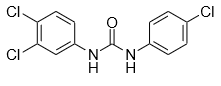 | Antisepsis | Anilide | (21) |
| Chlorhexidine | 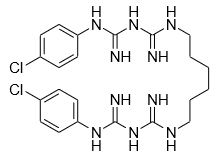 | Antisepsis, disinfection | Biguanide | (22,23) |
| Chloroxylenol | 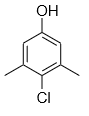 | Antisepsis, disinfection | Bisphenol | (24) |
| EDTA | 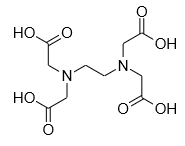 | Antisepsis, disinfection | Chelating agents | (25) |
| Triclosan | 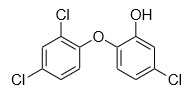 | Antisepsis | Halophenols | (26,27) |
| Silver nitrate | 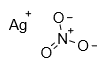 | Antisepsis, disinfection | Heavy Metal Derivatives | (28-33) |
| Sodium Hypochlorite | 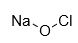 | Antisepsis,  disinfection | Halogen-releasing agent | (34) |
| Phenol | 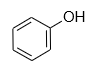 | Disinfection | Phenols and Cresols | (35-37) |
| Peracetic acid | 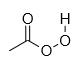 | Disinfection, sterilization | Peroxygens | (38) |
| Benzalkonium chloride (BAC) | 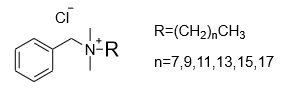 | Antisepsis, disinfection | Quaternary Ammonium Compound | (39,40) |
